# *Fgfr3* enhancer deletion markedly improves all skeletal features in a mouse model of achondroplasia

**DOI:** 10.1172/JCI184929

**Published:** 2025-01-16

**Authors:** Marco Angelozzi, Arnaud Molin, Anirudha Karvande, Ángela Fernández-Iglesias, Samantha Whipple, Andrew M. Bloh, Véronique Lefebvre

**Affiliations:** Department of Surgery, Division of Orthopaedic Surgery, Children’s Hospital of Philadelphia, Pennsylvania, USA.

**Keywords:** Bone biology, Genetics, Bone disease, Cartilage, Mouse models

## Abstract

Achondroplasia, the most prevalent short-stature disorder, is caused by missense variants overactivating the fibroblast growth factor receptor 3 (FGFR3). As current surgical and pharmaceutical treatments only partially improve some disease features, we sought to explore a genetic approach. We show that an enhancer located 29 kb upstream of mouse *Fgfr3* (*–29E*) is sufficient to confer a transgenic mouse reporter with a domain of expression in cartilage matching that of *Fgfr3*. Its CRISPR/Cas9-mediated deletion in otherwise WT mice reduced *Fgfr3* expression in this domain by half without causing adverse phenotypes. Importantly, its deletion in mice harboring the ortholog of the most common human achondroplasia variant largely normalized long bone and vertebral body growth, markedly reduced spinal canal and foramen magnum stenosis, and improved craniofacial defects. Consequently, mouse achondroplasia is no longer lethal, and adults are overall healthy. These findings, together with high conservation of *–29E* in humans, open a path to develop genetic therapies for people with achondroplasia.

## Abstract 2

By showing markedly improved skeletal features in achondroplasia mice upon deletion of an *Fgfr3* enhancer, this study paves a path for gene therapy in humans

## Graphical abstract

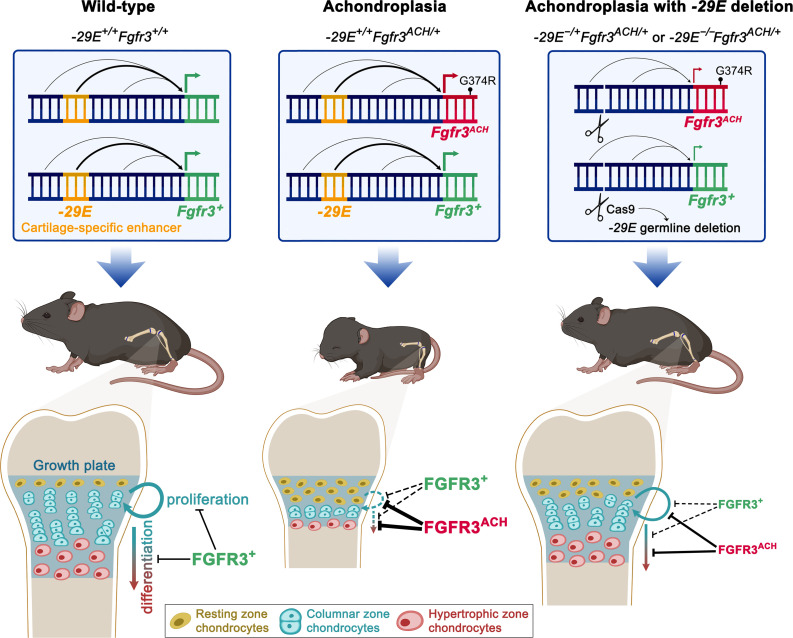

## Introduction

The fibroblast growth factor receptor 3 (FGFR3) is a tyrosine kinase receptor highly expressed in cartilage growth plates (GPs), where it restricts the main drivers of skeletal growth, including growth plate chondrocyte (GPC) proliferation and hypertrophic differentiation (1). Accordingly, *FGFR3* variants underlie several types of skeletal dysplasia. Loss-of-function variants cause a rare skeletal overgrowth syndrome typified by camptodactyly, tall stature and hearing loss (CATSHL; MIM 610474) (2, 3), while gain-of-function variants cause short stature conditions with appendicular, craniofacial, and axial skeleton defects (4). Ranging in severity from moderate to lethal, the latter conditions include hypochondroplasia (HCH; MIM 146000), achondroplasia (ACH; MIM 100800) and thanatophoric dysplasia (TD1; MIM 187600; and TD2; MIM 187606).

ACH is the most common form of short stature in humans (4.6 per 100,000 births (5)). About 98% of cases feature an autosomal-dominant, de novo, or inherited p.G380R variant, which over activates FGFR3 and renders it partially ligand independent (6, 7). Manifestations include mean height in the third percentile, macrocephaly with frontal bossing, midface hypoplasia, lumbar lordosis, rhizomelia, leg bowing, and brachydactyly with trident hand (8). They also include spinal canal and foramen magnum stenosis and upper airway obstruction, the neurological impacts of which — namely motor development delay, hydrocephalus, gait impairment, paraparesis or quadriparesis, myelopathy, and sleep apnea — reduce the quality of life and can be lethal (9). Long-term complications have also been described or suggested, such as chronic pain, obesity, diabetes, cardiovascular diseases (10, 11), and osteopenia (12, 13).

To date, ACH management is mostly symptomatic. Decompressive surgeries reduce spinal stenosis; tonsillectomy, turbinectomy, and adenoidectomy resolve sleep apnea; and limb lengthening procedures improve height and proportionality (14). However, these surgeries are not risk free and do not address all ACH features. Recent advances in understanding the molecular regulation of GPCs have suggested pharmacological options to target the interaction of FGFR3 with ligands, the FGFR3 tyrosine kinase domain, and downstream or parallel signaling pathways (15, 16). While most have had little success, the C-type natriuretic peptide (CNP) analogs Vosoritide and TransCon are showing promising results in clinical trials (17, 18), and Vosoritide has been approved for clinical use in several countries (19). However, limitations remain. For instance, Vosoritide improves the annualized growth velocity of preschool-aged children with ACH only partially (20), and its impact on final height and on spinal and cranial defects remains to be evaluated, together with long-term drug resistance and effects. Additionally, the treatment plan demands daily injections, which cause site reactions and hinder patient compliance. Thus, no current surgical or pharmacological approaches are entirely satisfactory, and there is an urgent need to develop new therapies that are safe and fully effective.

Gene therapy is a fast-evolving field, with strategies already approved for various diseases (21) but not for skeletal dysplasias. For ACH, variant correction is not currently feasible, given the impossibility of designing guide RNAs that uniquely recognize the mutant allele and the high risk that inactivating both *FGFR3* alleles by nonhomologous end-joining repair could cause CATSHL features. In this context, we investigated the option of lowering *FGFR3* expression substantially but not completely. We show that an enhancer located 29 kb upstream of *FGFR3* (*–29E*) contributed to the high expression of the gene in cartilage and that its deletion in an achondroplastic mouse model considerably mitigated all skeletal defects. Our findings help open the door to the development of genetic strategies to safely and effectively treat people with ACH and related conditions.

## Results

### FGFR3 expression is likely controlled by multiple enhancers.

**Figure 1:**
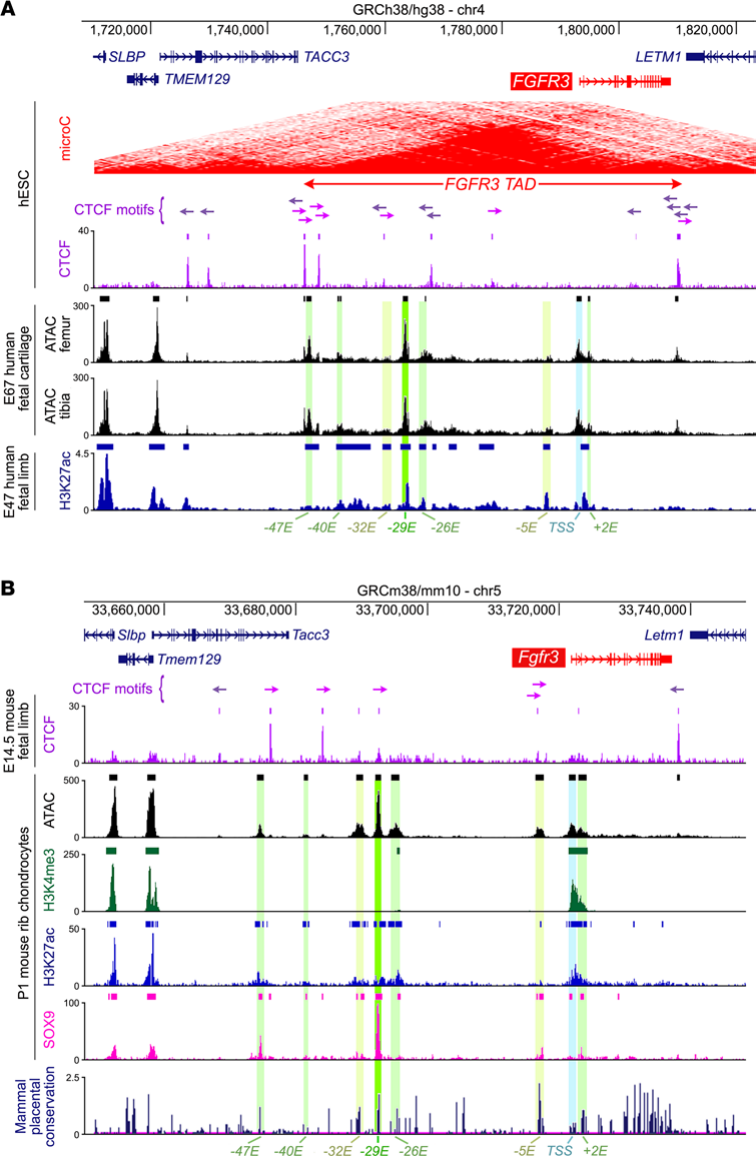
Identification of putative enhancers of human *FGFR3* and mouse *Fgfr3*. (**A**) From top to bottom, graphical representation of the human chromosome 4 segment containing *FGFR3* and neighboring genes; heatmap of chromatin folding from Micro Capture-C assay in H1-hESC cells (UCSC Genome Browser); and ATAC-seq and ChIP-seq data. For Micro Capture-C data, red color density reflects the number of interactions. For CTCF ChIP-seq data in H1-ESC cells (22), vertical lines and arrows indicate peaks and binding motif orientation, respectively. For ATAC-seq data in E67 human femoral and tibial fetal cartilage (24) and for H3K27ac ChIP-seq data in E47 human fetal limbs (25), vertical lines/blocks indicate peaks. Putative enhancers are highlighted in shades of green and the *FGFR3* transcription start site (TSS) in light blue. (**B**) Similar representation as in **A** but for mouse chromosome 5 segment encompassing *Fgfr3* and neighboring genes; CTCF ChIP-seq data in E14.5 fetal limbs (23); ATAC-seq data and ChIP-seq data for H3K4me3, H3K27ac, and SOX9 in P1 chondrocytes (26, 27), and histogram of placental mammal conservation scores (UCSC Genome Browser).

To locate enhancers that may direct *FGFR3* expression in cartilage, we mined available data on chromatin interactions and histone modifications at the human *FGFR3* locus and mouse and rat *Fgfr3* loci. Micro Capture-C assays in human embryonic stem cells (hESCs) showed *FGFR3* housed in a 63 kb topologically associating domain (TAD) separating it from its neighbors, *TACC3* and *LETM1* (Figure 1A). ChIP-seq in the same cells (22) and in fetal mouse limbs (23) for CTCF — which anchors chromatin at TAD boundaries — supported this finding (Figure 1, A and B). Assays for transposase-accessible chromatin–seq (ATAC-seq) and ChIP-seq for H3K27ac (histone modification marking active enhancers and promoters) in human fetal cartilage (24, 25), newborn mouse primary chondrocytes (26, 27), and rat chondrosarcoma (RCS) cells (28) revealed several transcriptionally active regions in this TAD (Figure 1, A and B and Supplemental Figure 1, A and B; supplemental material available online with this article; https://doi.org/10.1172/JCI184929DS1). The strongest peaks were seen at a sequence located 29 kb *(–29E*) upstream of the *FGFR3* transcription start site (TSS), followed by the TSS and a –*47E* sequence. Weaker peaks were seen 40, 32, 26, and 5 kb upstream (*–40E* to *–5E*) and 2 kb downstream (*+2E*) of the TSS. Several of these sequences, including *–29E,* but not the promoter region, are highly conserved in placental mammals (Supplemental Figure 1B). ChIP-seq revealed that the chondrogenic transcription factor SOX9 bound each putative enhancer in mouse chondrocytes and RCS cells (27, 28), with *–29E* displaying the strongest signal. *Fgfr3* expression in chondrocytes thus likely relies on *–29E* and several other enhancers, and the binding of these enhancers by SOX9 is consistent with the partial dependence of *Fgfr3* on SOX9 for expression in chondrocytes in vivo (29). Single-cell ATAC-seq in various *FGFR3*-expressing human fetal tissues (30) showed that chromatin was accessible at several sites in the *FGFR3* TAD, including *–40E*, *–32E*, *–26E*, and other regions, but not *–29E* (Supplemental Figure 2). We concluded that multiple enhancers may control *FGFR3* expression in cartilage and other tissues, and that *–29E* may be a leading cartilage-specific enhancer.

### –29E is a cartilage-specific enhancer.

**Figure 2:**
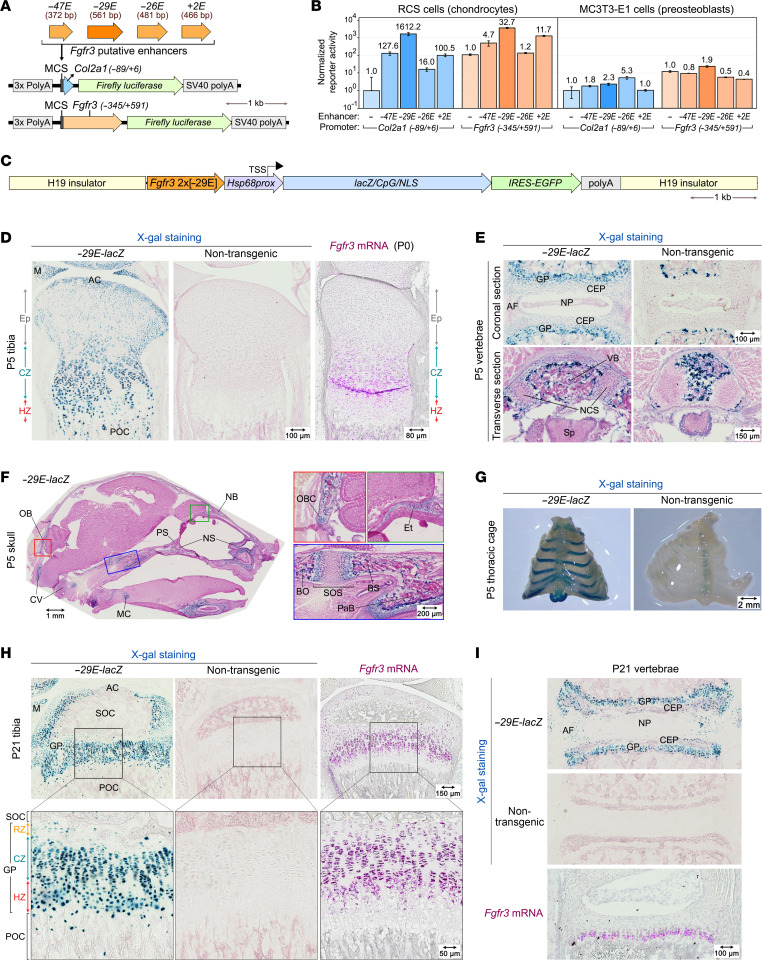
In vitro and in vivo assessment of *–29E* activity. (**A**) Reporters used in assays in vitro in **B**. Four putative enhancers were cloned in the multiple cloning site (MCS) of reporters driven by a *Col2a1* or *Fgfr3* promoter. polyA, polyadenylation sites. (**B**) Reporter activities assessed in transiently transfected RCS and MC3T3-E1 cells. Averages with SDs of firefly luciferase values normalized with NanoLuc values are shown for triplicate cultures from a representative experiment. Individual values are shown in the Supporting Data Values file. Numbers above bars indicate fold increases relative to promoter alone activities. (**C**) *–29E-lacZ* transgene flanked with H19 insulators. Two copies of *–29E* precede the *Hsp68* promoter (with transcription start site indicated), an optimized *lacZ* sequence, and IRES-EGFP and polyadenylation sequences. (**D**) Left, sections through the tibial proximal epiphysis of P5 *–29E-lacZ* and nontransgenic littermates stained with Xgal (blue) and counterstained with Nuclear Fast Red (pink). The position of the epiphysis (Ep), CZ, and HZ are indicated. Right, *Fgfr3* RISH of an equivalent section from a P0 WT mouse. The magenta color (RNA signal) was saturated and blue color (hematoxylin) desaturated using Adobe Photoshop. AC, articular cartilage; M, meniscus; POC, primary ossification center. Scale bars: 100 μm (X-gal staining images); 80 μm (right image). (**E**) X-gal staining of coronal and transverse sections through the vertebral column of same mice as in **D**. AF, annulus fibrosus; CEP, cartilaginous end plate; NCS, neurocentral synchondroses; NP, nucleus pulposus; Sp, spinal cord; VB, vertebral body. X-gal staining in ossification centers of transgenic and nontransgenic mice reflects endogenous β-galactosidase expressed in osteoclasts (66). Scale bars: 100μm (top); 150 μm (bottom). (**F**) X-gal staining of a sagittal section through the head of a P5 *–29E-lacZ* mouse. Colored-box areas in each image are shown at higher magnification on the right. BO, basioccipital bone; BS, basisphenoid bone; CV, cervical vertebrae; Et, ethmoid bone; MC, Meckel’s cartilage; NB, nasal bone; NS, nasal septum cartilage; OB, occipital bone; OBC, occipital bone cartilage; PaB, palatine bone; PS, presphenoid bone; SOS, spheno-occipital synchondrosis. Scale bar: 100 μm (left image) 200 μm (right image). (**G**) Whole-mount X-gal staining of the ventral portion of thoracic cages from same mice as in **D**. Scale bar: 2 mm. (**H**) Left, X-gal staining of tibia proximal epiphysis sections from P21 *–29E-lacZ* and nontransgenic littermates. Right, *Fgfr3* RISH of a P21 WT mouse section matching those used for X-gal staining. The image was processed as in **D**. Black-boxed areas are shown at higher magnification below. SOC, secondary ossification center. Scale bars: 150 μm (top); 50 μm (bottom). (**I**) Top, X-gal staining of vertebral column sections from the same mice as in **H**. Bottom, *Fgfr3* RISH of a P21 WT mouse section matching those used for X-gal staining. The image was processed as in **D**. Scale bars: 100 μm.

As a first step to functionally test the most promising *Fgfr3* enhancers, we cloned them into reporter plasmids upstream of a minimal *Col2a1* promoter (–89/+6) or the *Fgfr3* promoter region (–345/+591) (Figure 2A). We transfected the plasmids into RCS cells, which highly express *Fgfr3* as part of their GPC phenotype (31), and into preosteoblastic MC3T3-E1 cells, which express *Fgfr3* very weakly (Supplemental Figure 3A). In RCS cells, *–29E* potently activated both promoters, hinting that it does not require specific *Fgfr3* promoter elements to achieve potent transactivation in chondrocytes (Figure 2B). –*47E* and *+2E* were up to 10 times weaker than –29E, and –*26E* was even weaker. In MC3T3-E1 cells, none of the enhancers robustly activated transcription. These data further suggest that *–29E* could be a major, cartilage-specific enhancer of *Fgfr3*.

To test the activity of *–29E* in vivo, we constructed a reporter transgene (*–29E-lacZ*) containing 2 copies of *–29E*, the mouse *Hsp68* promoter (–664/+113), the *lacZ* (*E*. *coli* β-galactosidase) coding sequence and an internal ribosome entry site–EGFP (IRES-EGFP) cassette (Figure 2C). The *Hsp68* promoter was previously used in many transgenic reporters in mice (32). It was shown to be inactive by itself, but to be activated by enhancers from various types of genes (32). We flanked the reporter with mouse H19 insulator sequences to minimize genomic-site-of-integration effects (33). We obtained a transgenic mouse line and characterized it by X-gal (β-galactosidase substrate) staining. Staining of whole embryos at day 13.5 (E13.5) and of sections from E14.5 embryos showed transgene expression in all cartilage primordia and virtually nowhere else (Supplemental Figure 3, B and C). At postnatal day 5 (P5), *lacZ* was expressed in all and only cartilage tissues (Figure 2, D–G). Tibial proximal sections showed matching *Fgfr3* and transgene expression, both being stronger in columnar zone (CZ) and hypertrophic zone (HZ) GPCs than in articular and resting zone (RZ) chondrocytes. In the vertebral column, transgene activity was intense in endplate cartilage and weak in neurocentral synchondroses, annulus fibrosus, and nucleus pulposus (Figure 2E). In the head, Xgal staining marked the cartilage templates of endochondral bones, including the ethmoid and occipital bones, and cranial base synchondroses (Figure 2F). Rib and sternum cartilage also stained with X-gal (Figure 2G). Similar results were obtained at P21 and P56 (Figure 2, H and I and Supplemental Figure 3, D–F). Thus, *–29E* is sufficient to activate transcription in and only in the cartilage-specific domain of *Fgfr3* expression and it is active throughout embryonic and postnatal skeletal growth.

### –29E deletion robustly decreases Fgfr3 expression in vivo.

**Figure 3:**
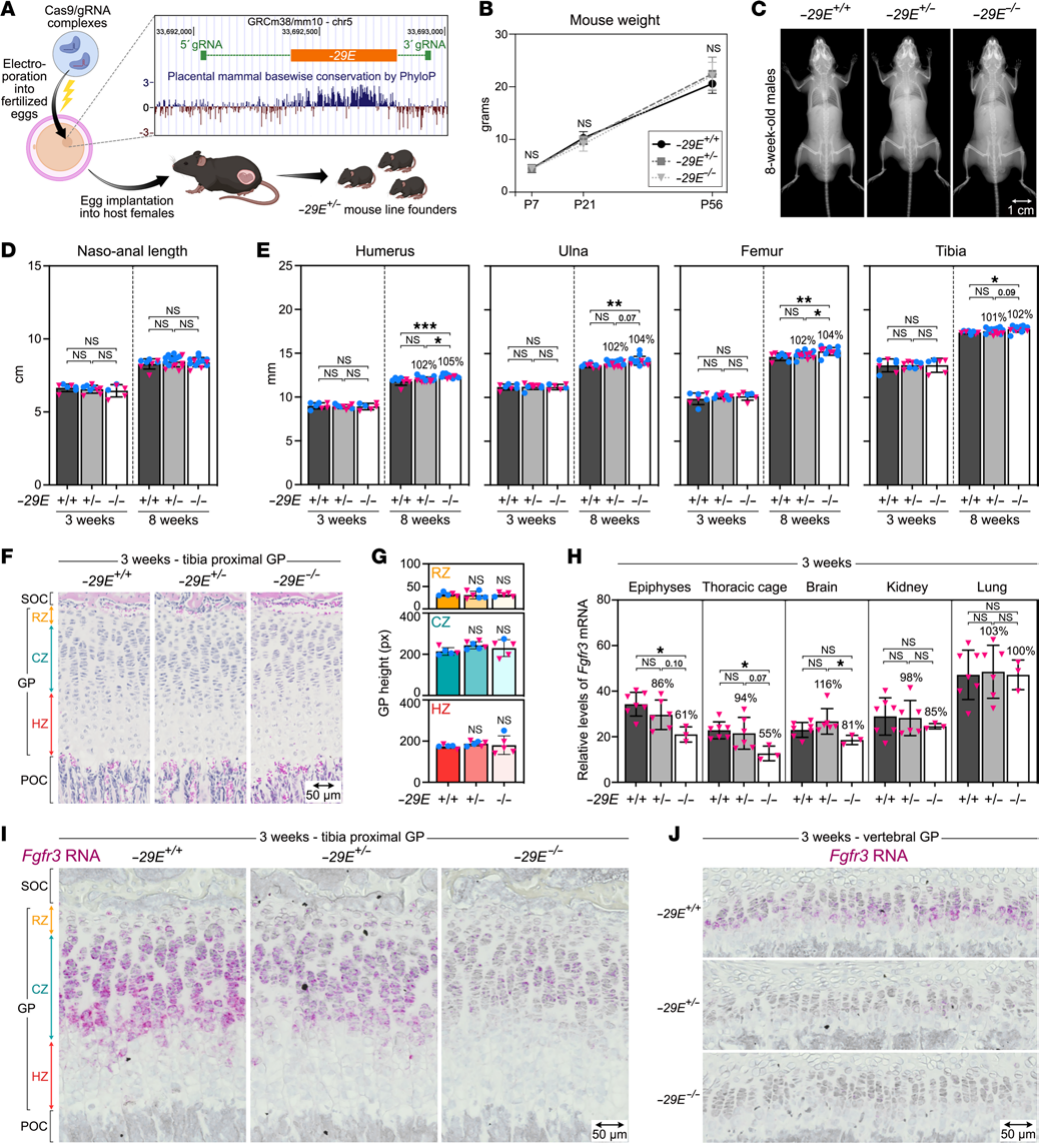
*–29E* deletion substantially lowers *Fgfr3* expression in cartilage without impacting health. (**A**) Strategy used to generate mice with *–29E* deletion. EP, electroporation. Inset, adapted UCSC genome browser representation of the mouse chromosome 5 segment containing *–29E* and the gRNAs used for *–29E* deletion. The plot shows placental mammal conservation of the enhancer compared with flanking sequences. (**B**) Weight curves of *–29E^+/+^*, *–29E^+/–^*, and *–29E^–/–^* mice (*n* = 5–17). Symbols and brackets represent means and SDs, respectively. Individual mouse weight values are shown in the Supporting Data Values file. Statistical analysis was performed in this panel and others using 1-way ANOVA followed by Tukey’s multiple comparison tests. No significant differences among genotypes were detected (ns). (**C**) X-rays of representative 8-week-old males. Similar results were obtained with females. Scale bar: 1 cm. (**D**) Naso-anal lengths of 3- and 8-week-old mice. Each symbol corresponds to a distinct mouse. Blue dots, males; pink triangles, females. (**E**) Long bone lengths of the same mice as in **D**. The percentages of average values obtained for *–29E^+/–^* and *–29E^–/–^* mice relative to *–29E^+/+^* mice are indicated at 8 weeks. *P ≤ 0.05; **P ≤ 0.01; ***P ≤ 0.001). *P* values slightly above significance are indicated. (**F**) Pictures of H&E-stained sections through tibial proximal GPs of representative 3-week-old mice. POC, primary ossification center; SOC, secondary ossification center. Scale bar: 50 μm. (**G**) Quantification of GP zone heights in the same group of mice (*n* = 5–6) as in **F**. No statistically significant difference (ns) was detected for mutant versus control GP zones. (**H**) RT-qPCR assays of *Fgfr3* RNA levels in skeletal and nonskeletal tissues. Only females were used. Percentages of average control values are indicated. (**I**) Representative *Fgfr3* RISH of sections through tibia proximal GPs. The magenta color (RNA signal) was saturated, and the blue color (hematoxylin) desaturated using Adobe Photoshop. Scale bar: 50 μm. (**J**) Representative *Fgfr3* RISH of sections through vertebral GPs. Images were processed as in **I**. Scale bar: 50 μm.

To assess the importance of *–29E* in the *Fgfr3* locus in vivo, we generated *–29E* knockout mice by electroporating CRISPR/Cas9 complexes in WT eggs (Figure 3A, and Supplemental Figure 4A). *–29E* deletion was confirmed in founder mice and their progeny by PCR and sequencing (Supplemental Figure 4, A–C). *–29E^+/–^* and *–29E^–/–^* mice looked healthy throughout life and had normal body weight and naso-anal length (Figure 3, B–D). Appendicular bones were of normal length at 3 weeks and they were 2%–5% longer and tended to have a slightly increased cortical and trabecular bone mass in 8-week-old *–29E^–/–^* mice compared with WT mice (Figure 3E and Supplemental Figure 4, D–G). GPs looked histologically normal (Figure 3, F and G).

RT-qPCR assays uncovered *Fgfr3* downregulation to 61% in long bone epiphyses and to 55% in thoracic cages of 3-week-old *–29E^–/–^* mice compared with controls (Figure 3H). Brain, lung, and kidney, which highly express *Fgfr3*, showed no significant expression change. RNA in situ hybridization (RISH) of appendicular and spinal sections showed substantial *Fgfr3* downregulation in GP and articular cartilage but not in periosteum and spinal cord (Figure 3, I and J and Supplemental Figure 5, A and B). *Tacc3*, *Letm1,* and *Tmem129*, which are *Fgfr3* neighbors, and *Sox9* and *Acan*, which are chondrocyte markers, were expressed normally (Supplemental Figure 5, C–E). These findings revealed that *–29E* strongly elevated *Fgfr3* expression in cartilage during postnatal growth but, since it is not absolutely required, its deletion could reduce ACH features without creating issues associated with complete expression loss in CATSHL.

### –29E deletion vitally improves skeletal features in achondroplastic mice.

We evaluated the effect of deleting *–29E* in an ACH mouse model. In this model, a loxP-flanked neomycin resistance cassette (*Neo*) is inserted in an *Fgfr3* intron, and a p.G374R variant matches the most common human ACH variant (p.G380R) (34). The *Neo* insertion renders the *Fgfr3^Neo^* allele nonfunctional, but its excision by Cre recombinase converts *Fgfr3^Neo^* into a genuine *Fgfr3^ACH^* allele. We deleted *–29E* in *Fgfr3^Neo/Neo^* eggs as we did for WT eggs, and generated experimental mice as follows (Supplemental Figure 6A). ACH (*Fgfr3^ACH/+^* or *–29E^+/+^Fgfr3^ACH/+^*) and control (*Fgfr3^+/+^* or *–29E^+/+^Fgfr3^+/+^*) mice were obtained as progeny of *Fgfr3^Neo/+^PrmCre* males (expressing Cre in the germ line [ref. 35]) and *Fgfr3^+/+^* females. *–29E^–/+^Fgfr3^ACH/+^* mice (lacking *–29E* in the *Fgfr3^ACH^* allele), *–29E^–/–^Fgfr3^ACH/+^* mice (lacking *–29E* in both *Fgfr3* alleles), and control mice (*–29E^+/+^Fgfr3^+/+^*) were obtained as progeny of *–29E^–/+^Fgfr3^Neo/+^PrmCre* males and *–29E^–/+^Fgfr3^+/+^* females.

**Figure 4:**
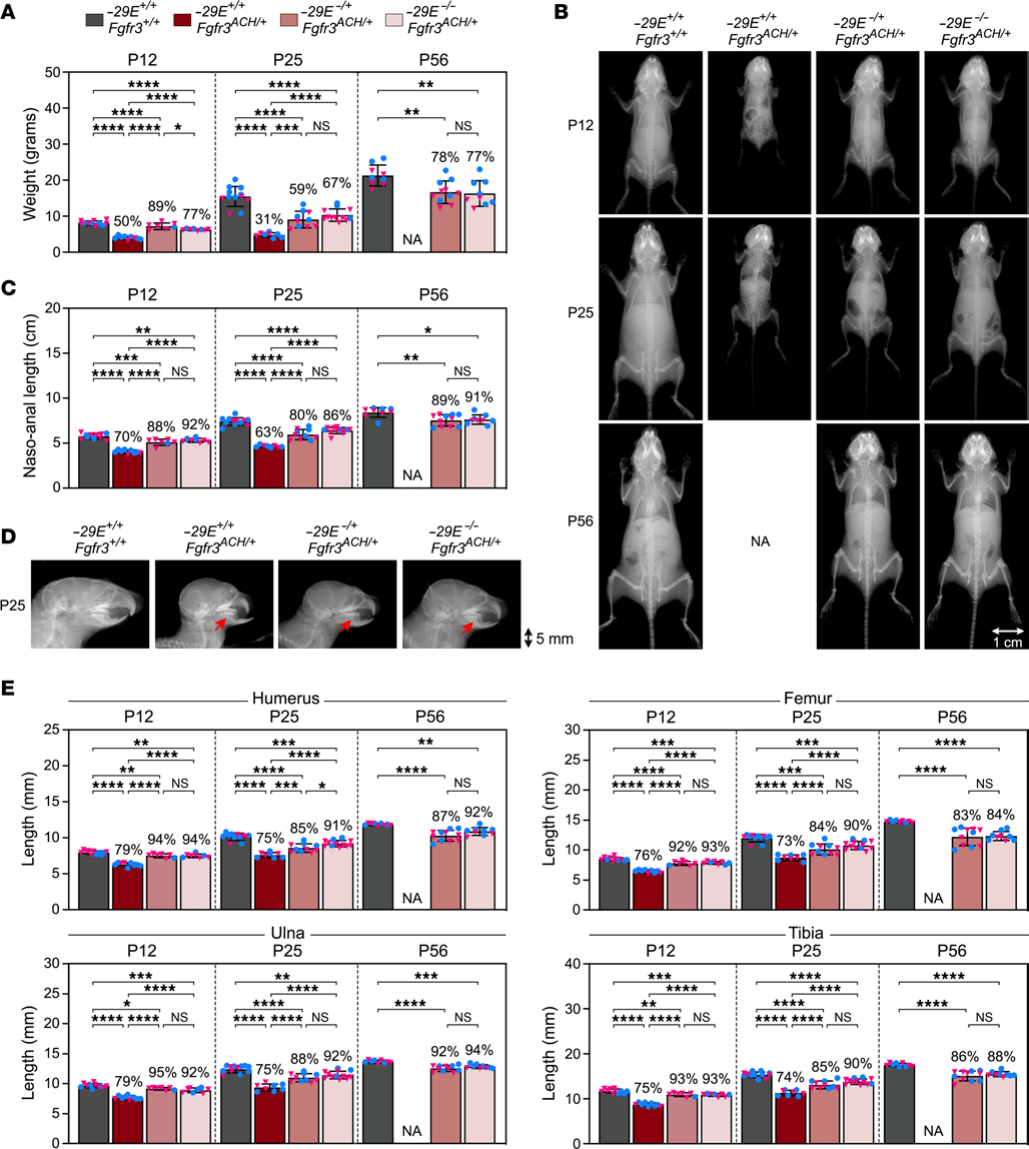
*–29E* deletion largely restores skeletal growth of achondroplastic mice. (**A**) Weights of WT mice, achondroplastic mice, and achondroplastic mice with *–29E* deletion in the ACH allele or both alleles at 3 ages. Bars and brackets represent means and SDs, respectively. Each symbol corresponds to a distinct mouse. Blue dots, males; pink triangles, females. The percentages of average values for each genotype group relative to WT mice are indicated. Statistical analysis was performed using 1-way ANOVA followed by Tukey’s multiple comparison tests. *P ≤ 0.05; **P ≤ 0.01; ***P ≤ 0.001; ****P ≤ 0.0001. NA, not available (*–29E^+/+^Fgfr3^ACH/+^* mice die around P25). (**B**) X-rays of the skeleton of representative mice. Scale bar: 1 cm. (**C**) Naso-anal lengths of mice from the same group as in **A**. (**D**) X-rays of the skulls of representative mice. Red arrows indicate jaw misalignment. Scale bar: 5 mm. (**E**) Long bone lengths of the same mice as in **A**.

*Fgfr3^ACH/+^* mice exhibited typical ACH features, irrespective of sex. They were indistinguishable from control littermates at birth, but by P12, their weight was 50% and their naso-anal length 70% of those of the controls (Figure 4, A–C). By P25, their condition had further worsened, their weight at 30% and their body length 65% of those of the controls. They had malocclusion due to uneven growth of the upper and lower jaws (Figure 4D) and thus needed incisor trimming to feed properly. Most died around that age, with aggravated skull vault doming suggesting hydrocephalus.

ACH mice did not improve when *–29E* was deleted in the WT allele only (*–29E^+/–^Fgfr3^ACH/+^* mice) (Supplemental Figure 6, B–E), but they did when *–29E* was deleted in the *Fgfr3^ACH^* allele (*–29E^–/+^Fgfr3^ACH/+^* mice) or in both *Fgfr3* alleles (*–29E^–/–^Fgfr3^ACH/+^* mice). Indeed, *–29E^–/+^Fgfr3^ACH/+^* and *–29E^–/–^Fgfr3^ACH/+^* pups had a weight and body length intermediate between that of control and ACH mice (Figure 4, A–C), jaw alignment improved (Figure 4D), and all reached adulthood. At P56, their weight was 77%–78% of that of the controls and their body trunk and appendicular bones were 83%–95% as long as controls (Figure 4, A–C and E). These numbers were comparable to those obtained in pups, indicating that the enhancer deletion likely had similar effects throughout postnatal growth. These mice looked healthy, ambulated normally, were fertile, and took good care of their pups (Supplemental Figure 6, F–H and Supplemental Videos 1–3).

*–29E* deletion also improved cortical and trabecular bone parameters of ACH mice (Supplemental Figure 7). At P25, the femur cortical bone area of ACH mice was 34% of that of control mice, mainly due to reduced cortical thickness (Supplemental Figure 7, A and B). The trabecular bone volume was 31%, as both trabecular number and thickness were decreased (Supplemental Figure 7, C and D). Bones were also slightly under mineralized (91%–95%). These features were fully or nearly normal in ACH mice with *–29E* deletion. Adult *–29E^–/+^Fgfr3^ACH/+^* and *–29E^–/–^Fgfr3^ACH/+^* mice tended to have thicker cortical bones, like *–29E^–/–^* mice. However, their trabecular bone mass was reduced by 39% and 52%, respectively, due to a lower number of trabeculae (Supplemental Figure 7, E–H). Since *–29E* is active only in chondrocytes, the resolution of osteopenia could originate from improved chondrogenesis or overall health condition. In sum, *–29E* deletion reduced the effect of ACH on skeletal growth at least by half, abolished lethality, lessened osteopenia, and critically improved mouse health from neonatal to adult ages.

### –29E deletion lessens skull and vertebral defects of achondroplastic mice.

**Figure 5:**
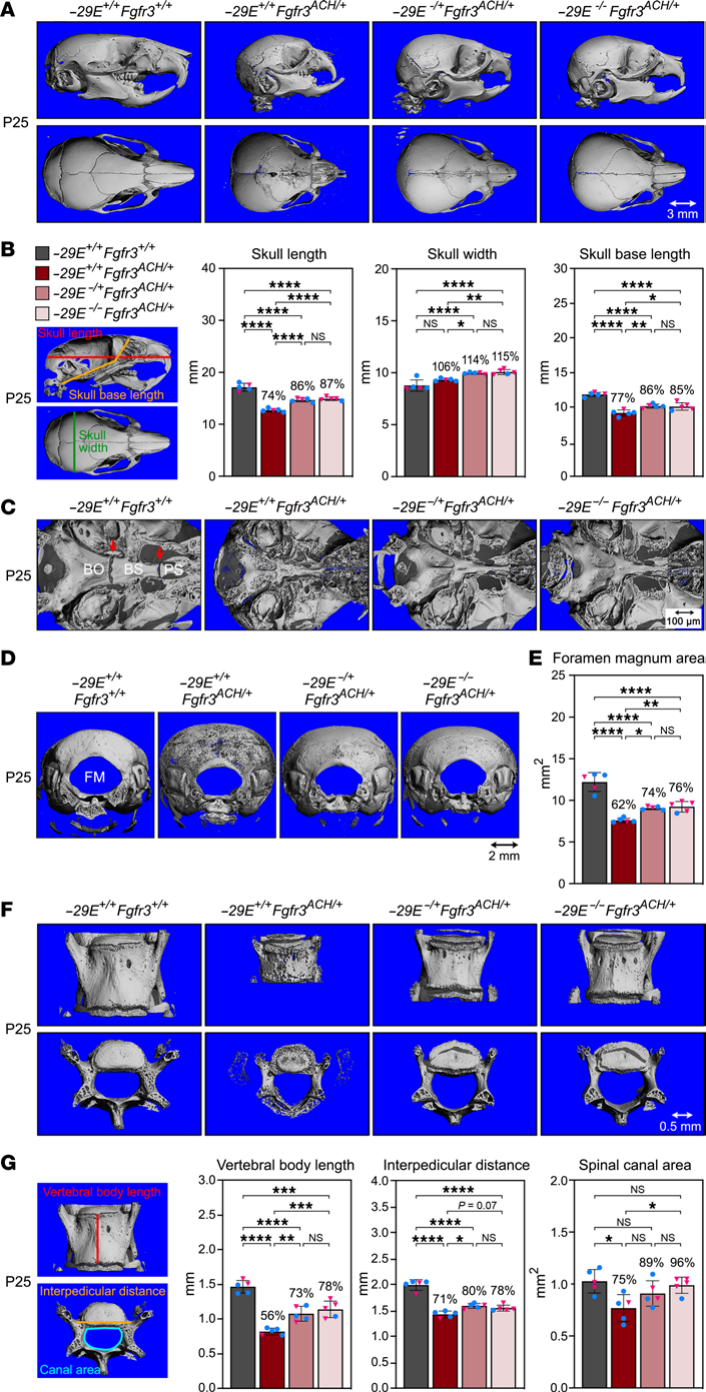
*–29E* deletion lessens skull and vertebral malformations of achondroplastic mice. (**A**) μCT reconstruction images of the skulls of representative P25 mice with the indicated genotypes. Top row, lateral view; bottom row, apical view. Scale bar: 3 mm. (**B**) Skull length, width, and base lengths of mice from same groups as those shown in **A**. Left, measurement schematic. Bars and brackets represent means and SDs, respectively. Each symbol represents a distinct mouse. Blue dots, males; pink triangles, females. The percentages of average values for each genotype group relative to WT mice are indicated. Statistical analysis was performed using 1-way ANOVA followed by Tukey’s multiple comparison tests. *P ≤ 0.05; **P ≤ 0.01; ***P ≤ 0.001; ****P ≤ 0.0001. (**C**) μCT reconstruction images of the skull base of representative mice. BO, basioccipital bone; BS, basisphenoid bone; PS, presphenoid bone. Red arrows, spheno-occipital and inter-sphenoid synchondroses. Scale bar: 100 μm. (**D**) μCT reconstruction images of the occipital area of skulls from representative mice showing the foramen magnum (FM). Scale bar: 2 mm. (**E**) Foramen magnum areas measured for the same mice as in **B**. (**F**) μCT reconstruction images of the L4 vertebrae from representative mice. Top row, coronal views; bottom row, transverse views. Scale bar: 0.5 mm. (**G**) Vertebral body length, interpedicular distance and spinal canal area measured for the L4 vertebrae of same mice as in **B**. Left, measurement schematic.

Since people with ACH have major axial and craniofacial malformations and since health normalization of *–29E*-deficient ACH mice unlikely resulted from appendicular and body trunk elongation, we examined the effect of *–29E* deletion on the skull and vertebrae. P25 *Fgfr3^ACH/+^* mice had prognathism, a dome-shaped skull, which was 26% shorter and 6% wider than that of controls, and a shortened cranial base (23%), in which synchondroses closed prematurely (Figure 5, A–C). The skull and cranial base lengths improved by half in *–29E^–/+^Fgfr3^ACH/+^* and *–29E^–/–^Fgfr3^ACH/+^* mice, whereas the skull was wider than in ACH mice (14%–15% relative to controls) and synchondroses still fused prematurely. The foramen magnum area of ACH mice was 62% that of the control mice, and *–29E* deletion improved it to approximately 75% (Figure 5, D and E). The skull length difference between control and *–29E^–/+^Fgfr3^ACH/+^* and *–29E^–/–^Fgfr3^ACH/+^* mice slightly worsened by adulthood, but the foramen magnum defect was stable (Supplemental Figure 8, A and B).

The vertebrae of P25 *Fgfr3^ACH/+^* mice had their height reduced to 56% of controls, their interpedicular distance to 71%, and their canal area to 75%, and bone was very porous (Figure 5, F and G). *–29E* deletion restored vertebral length to 73%–78%, interpedicular distance to 78%–80%, canal area to 89%–96%, and improved bone quality. By the time *–29E^–/+^Fgfr3^ACH/+^* and *–29E^–/–^Fgfr3^ACH/+^* mice reached adulthood, their vertebral parameters were virtually normal (90%–100%) (Supplemental Figure 8, C and D). Thus, *–29E* deletion substantially improved cranial and vertebral malformations of growing ACH mice and almost fully restored vertebral defects by adulthood. These findings likely reflected a continued beneficial effect of *–29E* deletion postnatally and were consistent with long-term survival of the mice, healthy appearance, and near normalization of body length by adulthood.

### –29E deletion improves GPs in achondroplastic mice.

**Figure 6:**
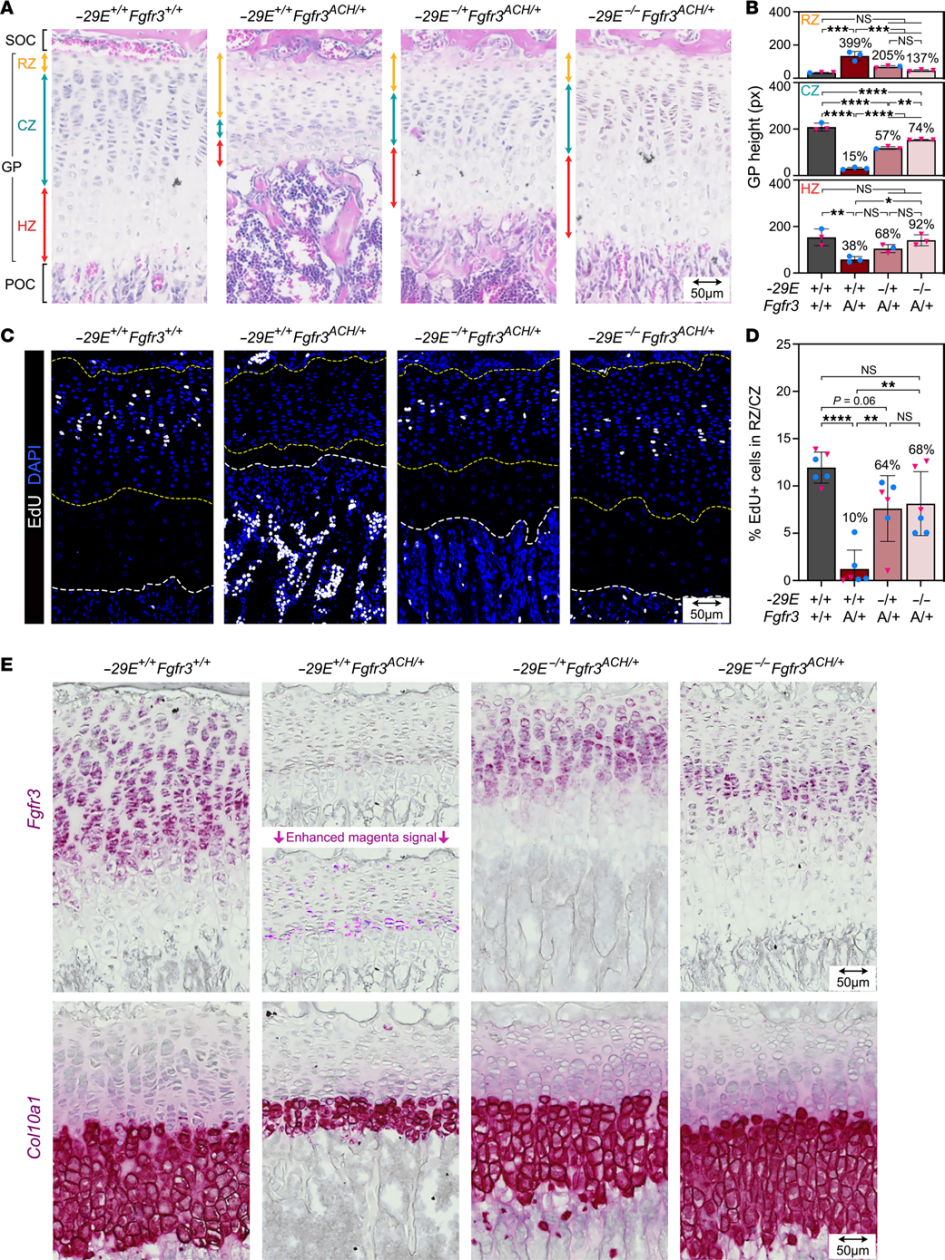
*–29E* deletion lessens GP defects in achondroplastic mice. (**A**) Pictures of H&E-stained sections of tibial proximal GPs of representative P25 mice with the indicated genotypes. POC, primary ossification center; SOC, secondary ossification center. Double arrows indicate GP zone heights. Scale bar: 50 μm.(**B**) Quantification of GP zone heights in mice (*n* = 3) from same groups as those in **A**. Each symbol corresponds to a distinct mouse. Blue dots, males; pink triangles, females. Bars and brackets represent means and SDs, respectively. Percentages are indicated for the average values obtained for each genotype group relative to WT mice. Statistical analysis was done using 1-way ANOVA followed by Tukey’s multiple comparison tests. *P ≤ 0.05; **P ≤ 0.01; ***P ≤ 0.001; ****P ≤ 0.0001. (**C**) Pictures of EdU incorporation assays in the same sections as in **A**. EdU signals are shown in white. Nuclei are counterstained with DAPI (blue). Dotted yellow lines demarcate RZ and CZ areas used for quantification. Dotted white lines mark the HZ bottom. Scale bar: 50 μm. (**D**) Percentages of EdU^+^ cells in the RZ/CZ zone of tibial GPs from the same groups as those in **C**. (**E**) *Fgfr3* and *Col10a1* RISH in sections adjacent to those in **A**. RNA signals are shown in magenta. The blue color (hematoxylin) was desaturated using Adobe Photoshop. The *Fgfr3* RNA signal obtained in ACH mice is shown before (top) and after (bottom) magenta signal saturation with Adobe Photoshop. Scale bars: 50 μm.

Histological analysis showed that tibial proximal GPs were almost half as short in P25 *Fgfr3^ACH/+^* mice as in controls (Figure 6, A and B and Supplemental Figure 9A). Based on cell morphology, the RZ (round chondrocytes) was estimated to be elongated fourfold in ACH mice, while the CZ (packed chondrocytes) and HZ (enlarged cells) were reduced to 15% and 38%, respectively. Deleting *–29E* in the ACH allele improved the GP height to 74% and deleting it in both *Fgfr3* alleles improved it to 86%. Each GP zone was ameliorated accordingly.

Consistent with these estimations, an EdU incorporation assay showed that GPC proliferation was virtually abolished in ACH mice and restored to 64% and 68% in *–29E^–/+^Fgfr3^ACH/+^* and *–29E^–/–^Fgfr3^ACH/+^* mice, respectively (Figure 6, C and D). RISH assays showed that *Fgfr3* mRNA was restricted to a thin CZ and was very low in ACH mice, suggesting activation of a negative feedback mechanism to reduce the impact of the overactive FGFR3 (Figure 6E and Supplemental Figure 9B). The mRNA regained abundance in *–29E^–/+^Fgfr3^ACH/+^* and *–29E^–/–^Fgfr3^ACH/+^* mice, but, as expected from the enhancer loss, its level was lower than in controls. RISH assays for *Col10a1* mRNA confirmed that the HZ was strongly shortened in ACH mice, partially recovered in *–29E^–/+^Fgfr3^ACH/+^* mice, and fully recovered in *–29E^–/–^Fgfr3^ACH/+^* mice (Figure 6E and Supplemental Figure 9B). Taken together, these data demonstrated that *–29E* deletion robustly mitigated the impact of ACH on GPCs.

### –29E deletion markedly restores FGFR3 signaling in achondroplastic mice.

**Figure 7:**
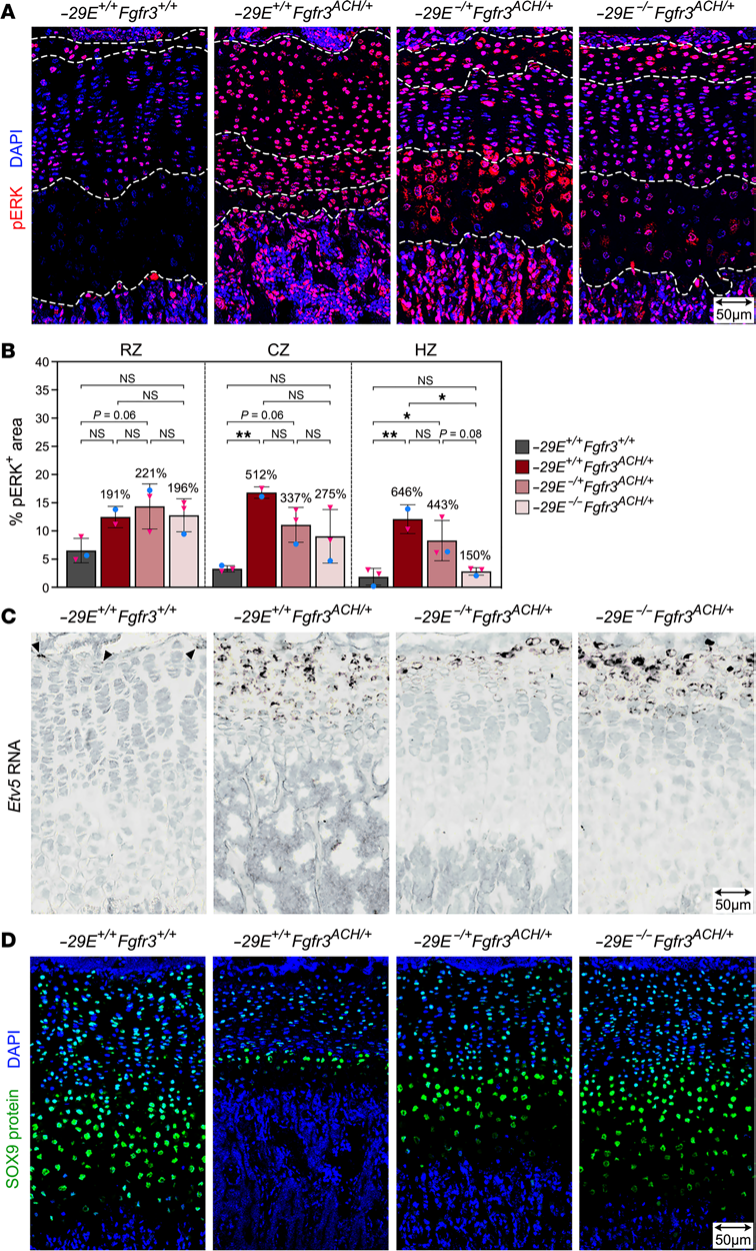
*–29E* deletion partially normalizes FGFR3 signaling in achondroplastic mice. (**A**) Pictures of representative pERK immunostaining assays in sections through tibia proximal GPs from representative P25 mice with the indicated genotypes. pERK signals are shown in red and nuclei in blue (DAPI). Dotted white lines demarcate the RZ, CZ and HZ. Scale bar: 50 μm.(**B**) Percentages of GP zone areas positive for pERK. Bars and brackets represent means and SDs, respectively. Percentages are indicated for the average values obtained for each genotype group relative to WT. Statistical analysis was done using 1-way ANOVA followed by Tukey’s multiple comparison tests. *P ≤ 0.05; **P ≤ 0.01. (**C**) *Etv5* RISH in sections from the same mice as in **A**. Arrowheads indicate *Etv5*^+^ cells in the WT RZ. The brown color (RNA) was saturated and blue color (hematoxylin) desaturated using Adobe Photoshop. Scale bar: 50 μm. (**D**) Pictures of representative SOX9 immunostaining assays in similar sections as in **A**. SOX9 signals are shown in green and nuclei in blue (DAPI). Scale bar: 50 μm.

To evaluate the extent of FGFR3 signaling restoration in ACH mice upon *–29E* deletion, we immunostained GPs for phospho-ERK1/2 (pERK), a key component and readout of the MAPK cascade activated by FGFR3. In control mice, most RZ and lower CZ GPCs exhibited a strong signal for pERK, whereas few upper CZ cells and almost no HZ cells showed a signal (Figure 7, A and B). In ACH mice, virtually all GPCs showed strong pERK staining, resulting in overall signals almost twice as high in the RZ and 5–6 times as high in the CZ and HZ. In *–29E^–/+^Fgfr3^ACH/+^* and *–29E^–/–^Fgfr3^ACH/+^* mice, pERK signals remained very high in the RZ but decreased by nearly half in the CZ. They were improved in the HZ of *–29E^–/+^Fgfr3^ACH/+^* mice and almost normalized in the HZ of *–29E^–/–^Fgfr3^ACH/+^* mice.

We consolidated these findings with RISH assays for *Etv5*, a transcriptional target of the RAS-MAPK pathway activated by FGF in development and cancer (36–38). In control mice, *Etv5* mRNA was detected only in a few RZ cells, whereas in ACH mice, it was abundant in all RZ cells and readily detectable in the entire CZ (Figure 7C and Supplemental Figure 9C). *Etv5* remained more expressed in *–29E^–/+^Fgfr3^ACH/+^* and *–29E^–/–^Fgfr3^ACH/+^* mice than in controls but was restricted to the RZ and upper CZ.

We also performed immunostaining assays for SOX9 because FGFR3-induced MAPK signaling was shown in vitro to increase SOX9 RNA and protein levels in chondrocytes, and this effect was exacerbated by the ACH mutation (39). Our in vivo data, however, revealed a decrease in SOX9 level in all GP zones of ACH mice. While differing from previous findings, likely because in vitro conditions do not fully recapitulate the in vivo complexity, they are consistent with impaired chondrocyte differentiation at all stages of maturation and with striking clinical similarities between ACH and Campomelic Dysplasia, which is due to *SOX9* haploinsufficiency (40, 41). Like other defects, this decrease in SOX9 level was substantially overcome with *–29E* deletion (Figure 7D). Altogether, these results indicated that *–29E* deletion normalized FGFR3 signaling only partially, but enough to largely restore key GPC activities, including proliferation and hypertrophy.

## Discussion

This study investigated the efficacy and safety of reducing FGFR3 expression as a possible treatment for ACH. It identified a *–29E* enhancer contributing to about half of the gene expression in mouse cartilage. Its germline deletion appeared harmless in otherwise WT mice, but improved all skeletal manifestations of mice modeling human ACH in what we believe to be an unprecedented manner. This enhancer is highly conserved in humans, where epigenetic data indicates that it is active in cartilage too. These findings pioneer exploration of therapies that would target enhancers or would otherwise reduce gene expression in ACH and other disorders due to gain-of-function variants.

We focused on *–29E* because chromatin accessibility, epigenetic modification, and transcription factor occupancy data in vivo and in vitro indicated that it could be the main *FGFR3* enhancer in cartilage. Our interest in a cartilage-specific enhancer was based on the premise that all major ACH manifestations result from FGFR3 over activation in chondrocytes and that a chondrocyte-specific genetic modification could carry a lower risk of long-term adverse effects than a global modification. We validated the cartilage specificity of *–29E* by showing that it was sufficient to activate a reporter in all and only cartilaginous tissues in transgenic mice tested from early fetal to adult ages. Like *Fgfr3* expression, it was stronger in CZ GPCs and weaker in RZ and articular chondrocytes. Matching our findings, another group reported that *–29E* drove transgene expression in all cartilages of E14.5 mouse embryos (only age analyzed) (42). *–29E* activity overlapped with but did not fully match that of other genes’ cartilage enhancers (42–46). This can be explained by the dependence of all enhancers on SOX9 but likely also on other factors specifying differential activities. By knocking out *–29E*, we demonstrated its contribution to about half of *Fgfr3* expression in cartilage and its lack of activity in other tissues and in regulating neighboring genes. This *Fgfr3* downregulation resulted in slightly longer limb bones in otherwise WT mice. No health issue was apparent, but in-depth phenotyping would be needed to fully rule this out. For instance, mice might develop premature osteoarthritis (OA), since a previous study showed that *Fgfr3* inactivation in chondrocytes led to this outcome (47). However, *Fgfr3* over activation by an ACH variant protected mice against the condition and, likewise, humans with ACH exhibit lower OA incidence (48). We thus postulated that the benefits of *–29E* inactivation in people with ACH could outweigh the risks.

We substantiated this prediction using a faithful preclinical model, wherein mice (*Fgfr3^ACH/+^*) harbor an *Fgfr3*-knockin variant (p.G374R) orthologous to the most common human ACH variant (p.G380R) (34). Excising the enhancer from the WT *Fgfr3* allele did not lead to significant improvement, but excising it from the ACH allele was very effective, and excising it from both alleles was even better. This differential allele effect reflects the over activity of the ACH versus WT receptor. The shortness of vertebral bodies and spinal canal stenosis were almost fully abolished in *–29E^–/+^Fgfr3^ACH/+^* and *–29E^–/–^Fgfr3^ACH/+^* mice, and the foramen magnum stenosis was greatly improved. These outcomes are very encouraging for clinical translatability, since most neurological issues in ACH are due to nerve compression resulting from spinal canal and foramen magnum stenosis. In addition to being painful, these defects can be lethal. Decompression surgeries are often performed to lessen stenosis, but these invasive procedures cannot be done for all vertebrae. A genetic treatment improving all vertebral and foramen magnum defects could thus greatly increase the quality of life and survival rate of people with ACH. Appendicular bones of *–29E^–/+^Fgfr3^ACH/+^* and *–29E^–/–^Fgfr3^ACH/+^* mice attained a length intermediate between that of ACH and WT mice. In humans with ACH, this outcome, together with vertebral column elongation, would result in a substantial height gain without requiring multiple rounds of limb lengthening surgeries. Craniofacial defects were improved but not fully corrected in *–29E^–/+^Fgfr3^ACH/+^* and *–29E^–/–^Fgfr3^ACH/+^* mice. Skull shortening and jaw misalignment in ACH mice, like midface hypoplasia with jaw misalignment and flat nasal bridge in humans with ACH, are mostly due to cranial synchondrosis premature fusion (49), which was not fully avoided by *–29E* deletion. Likewise, our genetic approach could only partially resolve craniofacial defects in individuals with ACH. It is possible that the bidirectional organization of cranial synchondroses (49) makes their stem cells (yet to be identified) more vulnerable to overactive FGF signaling than long bone GP stem cells. The skull vault was widened in *Fgfr3^ACH/+^* mice, likely due to cerebrospinal fluid excess (50, 51). Intriguingly, it was even wider in *–29E^–/+^Fgfr3^ACH/+^* and *–29E^–/–^Fgfr3^ACH/+^* mice. This may be a compensatory feature deployed more efficiently in these mice to mitigate the deleterious effects of cerebrospinal fluid excess and ensure mouse survival. Of note, the increased longevity of *–29E^–/+^Fgfr3^ACH/+^* and *–29E^–/–^Fgfr3^ACH/+^* mice compared with ACH mice means that they could constitute a helpful model to test the long-term safety and efficacy of complementary drug treatments.

Tissue, cell, and molecular analyses showed that *–29E* deletion markedly improved all activities of ACH GPCs. The RZ expansion and the CZ/HZ depletion, which were considerable in *Fgfr3^ACH/+^* mice, were negated by more than half in *–29E^–/+^Fgfr3^ACH/+^* and *–29E^–/–^Fgfr3^ACH/+^* mice. Although there are conflicting reports on the effect of FGFR3 and its ACH variant on the expression of such key paracrine factors as Indian hedgehog and parathyroid hormone-related protein (52–54), it is likely that ACH impacts GPCs, in part, through noncell-autonomous effects. However, evidence that *Fgfr3* is expressed in all GPCs and that ACH mice weightily increased the level of pERK in all GP zones strongly suggests that FGFR3 and its ACH variant also have key cell-autonomous effects in each zone. Noticeably, *–29E* deletion appeared to mitigate these effects less effectively in the RZ than CZ and HZ, as *–29E^–/+^Fgfr3^ACH/+^* and *–29E^–/–^Fgfr3^ACH/+^* mice maintained high pERK levels specifically in the RZ and high expression of *Etv5*, a readout of MAPK signaling. RZ cells might be particularly responsive to FGFR3 activation, such that the remaining elevated level of variant receptor was sufficient for maximal signaling. Elevated MAPK signaling in ACH was proposed to inhibit GPC hypertrophy in one study (55) and proliferation in another (56). The threshold of overactivity of MAPK and other signaling pathways needed to severely impair GPC activities is unknown, but our findings demonstrated that, although the *–29E* deletion did not fully normalize FGFR3 signaling, it lowered it enough for GPCs to resume effective proliferation and hypertrophy.

By establishing *–29E* as a key, but not absolute, enhancer for *Fgfr3* expression in cartilage, our findings imply that additional enhancers participate in driving *Fgfr3* expression in cartilage and other tissues. The Ornitz group previously proposed that the promoter region was sufficient to activate a transgene in several *Fgfr3*-expressing tissues (cartilage was not tested) and that a –2 kb enhancer was active in chondrocytes in vitro (57, 58). We identified several candidates at more distal locations, and although none were as active as *–29E* in reporter assays in RCS cells in vitro, we cannot rule out critical roles in vivo. Their positions in the *FGFR3* locus may influence their effects. Also, unlike *–29E*, they may be more active in RZ and upper CZ GPCs than lower CZ cells, explaining their low performance in RCS cells (lower CZ GPCs) and the poor ability of *–29E* deletion to lower FGFR3 signaling in ACH RZ cells. Alternatively, they may be more active in specific skeletal elements, such as craniofacial ones, where *–29E* deletion did not have as much effect as in appendicular and vertebral GPs. New studies are thus warranted to test these enhancers and promoter elements, identify their main cis-acting elements and transacting factors, and, thereby, to better understand the modular regulation of *FGFR3* in cartilage and other tissues. Of note, we searched the Open Targets Database for expression quantitative trait loci (eQTLs) at *–29E* that have been associated with *FGFR3* expression and human height or skeletal malformations, but did not obtain positive hits. Possible explanations are that eQTL studies did not use cartilage samples and that discrete variants at this enhancer do not impact *FGFR3* expression strongly enough to cause noticeable phenotypes.

We believe that the results of the present study represent a major translational research step forward by showing that genetic strategies reducing *FGFR3* expression could effectively improve all skeletal features of people with ACH and related conditions. The correction of the single-nucleotide ACH variants by CRISPR technology is the most logical of all conceivable strategies. However, it carries a high risk of generating null alleles by nonhomologous repair of DNA breaks, and since no gRNAs can be designed to target the ACH allele but not the WT allele, attempts to correct the ACH allele could create features of CATSHL, a severe syndrome caused by homozygous inactivation of *FGFR3*. The present study offers attractive alternatives, i.e., to delete the cartilage-specific *–29E* *FGFR3* enhancer (CRISPRko) or to block its activity (e.g., CRISPRi). It also infers that reducing *FGFR3* expression by any other means (e.g., siRNA-mediated knockdown) could be successful. These options will become possible upon identification of a vehicle (e.g., adeno-associated virus or nanoparticle) that can penetrate the avascular, dense, and highly charged cartilage extracellular matrix and thereby deliver the therapeutic reagents to chondrocytes. Many laboratories are working on designing such vehicles for articular cartilage and their promising results give hope that success is imminent for both articular and GP cartilage tissues (59, 60). Enhancer deletion would, in principle, have to be achieved only once in GP stem cells, since it would be transmitted to all GP daughter cells. According to current models (61, 62), these cells reside at the top of GPs, close to blood vessels, and should therefore be easier to reach than chondrocytes located deeper in cartilage tissue. In contrast, strategies to lower gene expression would require prolonged treatment. Since skeleton patterning and growth start in early fetuses and continue until puberty, treatment should preferably be initiated in utero to obtain maximum effects, but they would likely provide welcome benefits if started neonatally or at any age before puberty. In addition to timing, other determinants of success would be the level of *FGFR3* downregulation achieved and the percentage of GP stem cells or chondrocytes targeted; indeed, enhancer interference may not be as efficient as enhancer deletion and mosaic targeting could cause additional skeletal deformities. Besides being used as a genomic target, *–29E* could be instrumental as a tool to direct expression of therapeutic reagents (e.g., Cas9 and gRNAs) to chondrocytes only. This specificity could be cardinal to reduce the risks of potential side effects and to avoid germline transmission of the induced genetic modification.

In conclusion, this study has given proof-of-principle demonstration that strategies leading to *FGFR3* downregulation, which, to our knowledge, have not been attempted yet, could improve all skeletal features of ACH and related conditions and thus achieve superior outcomes than currently approved treatments. Moreover, it has identified an *FGFR3* cartilage-specific enhancer (*–29E*) as a possible tool and target for such therapeutic interventions.

## Methods

### Sex as a biological variable.

Our study examined male and female animals. Because sex was not found to affect the ACH phenotype, we analyzed males and females together and ensured that each sex was similarly represented among groups.

### In silico analysis of putative FGFR3 enhancers.

Putative *FGFR3* enhancers were identified by mining the following data: Micro Capture-C assay in H1-hESC cells and mammal placental conservation scores from the UCSC Genome Browser (https://genome.ucsc.edu); single-cell ATAC-seq assays in nonskeletal human fetal cells from the DESCARTES database (https://descartes.brotmanbaty.org); and other data from the NCBI Gene Expression Omnibus repository at accession numbers GSE116862 (CTCF ChIP-seq in H1-hESC cells), GSE153260 (ATAC-seq in human fetal cartilage), GSE42413 (H3K27ac ChIP-seq in human fetal limbs), GSE29184 (CTCF ChIP-seq in mouse fetal limbs), GSE178293 and GSE69109 (ATAC-seq and ChIP-seq assays in mouse rib chondrocytes), and GSE70144 (ChIP-seq assays in RCS cells). Data were analyzed in Galaxy (https://usegalaxy.org) using bowtie2 for alignment to reference genomes (63) and MACS2 for peak calling (64). CTCF motifs were identified using FIMO (65) in the MEME suite (https://meme-suite.org/meme/index.html). Data were visualized using Integrative Genomics Viewer (66).

### Reporter plasmid construction and assay.

The *Fgfr3* proximal region (–345/+591) and putative enhancers were PCR amplified from C57BL/6J mouse genomic DNA using Phusion High-Fidelity DNA Polymerase (New England Biolabs) and primers listed in Supplemental Table 1. Enhancers were cloned in p89Luc upstream of a minimal *Col2a1* promoter (–89/+6) and the firefly luciferase sequence (67). They were also cloned in *pFgfr3prox-Luc*, obtained by replacing the *Col2a1* promoter in p89Luc. The integrity of all inserts was verified by Sanger sequencing. Plasmids were cotransfected with the pNL1.1.TK[Nluc/TK] (NanoLuc) control reporter in RCS (RRID:CVCL_S122) and MC3T3-E1 (CRL-2593, ATCC; RRID:CVCL_5440) cells using Lipofectamine 3000 (Thermo Fisher Scientific). RCS cells were cultured in DMEM (Thermo Fisher Scientific) with 10% FBS (Sigma-Aldrich), and MC3T3-E1 cells in α-MEM (Thermo Fisher Scientific) with 10% FBS and no ascorbic acid. Cells were plated in 12-well dishes (1 × 10^5^ cells/well) and transfected 4 hours later. Cell extracts were made the next day in 0.1 M potassium phosphate buffer (pH 7.8) with 0.2% Triton X-100 (Thermo Fisher Scientific). Reporter activities were measured using Nano-Glo Dual-Luciferase Reporter Assay (Promega) and GloMax Explorer Microplate Reader (Promega). Reporter activities were normalized for transfection efficiency using NanoLuc values.

### Mice.

All mouse lines were kept on a C57BL/6J background and housed in standardized conditions to avoid intergroup variation. They carried a *–29E*-*lacZ* transgene (see below) or a combination of a *PrmCre* transgene (35) and *Fgfr3^+^*, *Fgfr3^Neo^* (conditional ACH; RRID:MGI:3639743) (34), and *Fgf3^ACH^* (ACH) alleles with or without *–29E* deletion (see below). They were genotyped by PCR using KAPA HotStart Mouse Genotyping Kit (Sigma-Aldrich) and specific primers (Supplemental Table 2). The incisors of *Fgfr3^ACH/+^* mice were trimmed as needed (once or twice a week) and moistened food pellets were provided on the cage floor.

### Generation of –29E-lacZ transgenic mice.

The *–29E*-*lacZ* transgene was built in pWHERE (InvivoGene). This plasmid provides H19 insulator sequences at both transgene ends, a multiple cloning site, a CpG-free *lacZ* sequence, including a nuclear localization signal to facilitate transgene expression and detection, and polyadenylation sites. We inserted the *Hsp68* proximal region (*Hsp68prox*, –664/+113) 5’ of *lacZ* to serve as promoter (46, 68, 69)and an IRES-EGFP cassette 3’ of *lacZ* (70). The *–29E* sequence was PCR-amplified from C57BL/6J mouse DNA using the same primers as for reporters (Supplemental Table 1). Two tandem copies were cloned upstream of *Hsp68prox*. The transgene was verified by Sanger sequencing and excised from the plasmid backbone using PacI. Fertilized eggs were microinjected with the transgene and implanted into host females. A founder male and his progeny were identified by *lacZ* PCR genotyping. Primers are listed in Supplemental Table 2.

### Generation of –29E^+/–^ mice.

Mice with *–29E* deletion in *Fgfr3^+^* and *Fgfr3^Neo^* were generated using the CRISPR/Cas9 approach and gRNAs with high on-target and low off-target scores (Supplemental Table 3). Cas9/gRNA/tracrRNA mixtures were electroporated into fertilized *Fgfr3^+/+^* and *Fgfr3^Neo/Neo^* eggs. Eggs that retained normal morphology were transferred into surrogate mothers. Founder mice and progeny were identified by PCR. Sanger sequencing of PCR products was performed to confirm *–29E* deletion. We amplified 2 independent mouse lines and obtained identical phenotypes. We describe findings for one line.

### X-gal staining.

*–29E-lacZ* expression was characterized by staining with X-gal (Thermo Fisher Scientific), a colorimetric substrate of β-galactosidase (*lacZ* product). X-gal staining of whole embryos and tissue sections was done following standard protocols (70). Tissues were collected after euthanasia, fixed in PBS-T (phosphate-buffered saline with 0.1% Tween 20 [Thermo Fisher Scientific]) with 2% paraformaldehyde (PFA [Electron Microscopy Sciences]) and 0.2% glutaraldehyde (Sigma-Aldrich) for 2 hours at room temperature and demineralized in 15% EDTA (Thermo Fisher Scientific), pH 7.4, for 4 days at 4°C. Samples were then immersed sequentially in 15% and 30% sucrose-PBS until sinking and embedded in O.C.T. medium (Thermo Fisher Scientific). 7- to 12-μm frozen sections were obtained and covered with X-gal solution (1 mg/mL X-gal in 0.1 M sodium phosphate buffer, pH 7.3 containing 2 mM MgCl_2_, 0.01% sodium deoxycholate, 0.02% Igepal CA-630, 20 mM Tris, 5 mM K_3_Fe(CN)_6_ and 5 mM K_4_Fe(CN)_6_ [Thermo Fisher Scientific]) for 5 hours or overnight at 37°C. Sections were counterstained with Nuclear Fast Red and mounted using ProLong Gold Antifade Mountant (Thermo Fisher Scientific). Slides were imaged using ZEISS Axio Scan.Z1 scanner. The H19 insulator sequences caused imprinting, such that progeny from transgenic males, but not females, expressed *–29E-lacZ*. X-gal staining corresponding to endogenous β-galactosidase produced by osteoclasts is seen in bone tissue in some sections.

### Radiography and microcomputed tomography analysis.

Radiographs of mice were acquired postmortem using a Faxitron X-ray System at 50 kV for 5 seconds. Bone length was measured on contact sides using lateral view images and built-in software. Data are reported as averages obtained for left and right limbs. For microcomputed tomography (μCT), legs, spine and heads were fixed in 4% PFA in PBS for 48 hours at room temperature. Analyses were performed using a SCANCO μCT 45 scanner (SCANCO Medical AG). Bones were imaged with an X-ray tube voltage of 55 kV and current of 145 μA, with a 0.5-mm aluminum filter. For femurs and vertebrae, voxel size was 4.5 μm and integration time was 400 milliseconds. For skulls, voxel size was 12.2 μm and integration time was 200 milliseconds. Cross-sectional images were reconstructed with built-in software. Trabecular and cortical bone analyses of femurs were done on distal metaphyses. For trabecular bone, a volumetric region of interest was defined starting 50 layers (225 μm) below the GP end and extending 200 layers distally (900 μm thick). For cortical bone, a slice of the diaphysis was defined starting 600 layers (2.7 mm) below the GP end and extending for 100 layers distally (450 μm thick). 3D reconstructions of L4 vertebrae and skulls were done using built-in software. Measurements were done on reconstructed images using ImageJ (https://imagej.net/ij/index.html).

### Histology, RNA in situ hybridization, and immunostaining.

For assays on sections, legs and spines were fixed as described above and demineralized in Morse’s solution for 48 hours at room temperature (71). Samples were dehydrated, embedded in paraffin, and 7-μm sections were obtained. Staining with H&E was done following a standard protocol. RNA in situ hybridization (RISH) was performed using RNAscope 2.5 HD detection reagent kit-RED or -BROWN and probes for mouse *Col10a1* (426181), *Etv5* (316961), and *Fgfr3* (444101) (Advanced Cell Diagnostics) (72). Slides were imaged using ZEISS Axio Scan.Z1 scanner. Cell proliferation was assessed by EdU (5-ethynyl-2’-deoxyuridine) incorporation using the Click-iT EdU Alexa Fluor 488 Imaging Kit (Thermo Fisher Scientific) (73). Mice were injected intraperitoneally with 250 μg EdU /10 g body weight 2 hours before euthanasia. For pERK and SOX9 immunostaining, sections were deparaffinized and antigen retrieval was performed with citrate buffer pH 6.0 for 2 hours at 60°C followed by 0.5% Triton X-100 in Tris-buffered saline (TBS) for 5 minutes at room temperature. Blocking was performed using 10% goat serum (Vector Laboratories) in TBS with 0.1% Tween 20 for 30 minutes at room temperature. Proteins were detected using a monoclonal anti-pERK antibody (dilution 1:200; 9101, Cell Signaling; RRID:AB_331646) and a biotinylated anti-rabbit IgG antibody (dilution 1:500; BA-1000, Vector Laboratories; RRID:AB_2313606) or a polyclonal anti-SOX9 antibody (dilution 1:500; AB5535, Sigma-Aldrich; RRID:AB_2239761) and an Alexa Fluor 488-conjugated anti-rabbit IgG antibody (dilution 1:500; A-11008, Thermo Fisher Scientific; RRID:AB_143165). Signals for pERK were amplified with TSA-Fluorescein kit (Akoya Biosciences). Slides were counterstained with DAPI and mounted using ProLong Gold Antifade Mountant. Images were acquired using a Leica TCS SP8 confocal microscope. Measurements were done using ImageJ software.

### Quantitative reverse transcription PCR.

For RNA extraction, tissues or cells were collected in TRIzol (Thermo Fisher Scientific) and dissociated using a PowerGen 125 Homogenizer (Thermo Fisher Scientific). For knee epiphyses (distal femur and proximal tibia) and thoracic cages (sternum and ventral portion of rib cage), total RNA was obtained by chloroform extraction followed by RNA mate (BioChain). For other samples, total RNA was extracted using RNAeasy kit (Qiagen). RNA was quantified using NanoDrop 2000 (Thermo Fisher Scientific) and 500 ng was used for reverse transcription with High-Capacity cDNA Reverse Transcription Kit (Thermo Fisher Scientific). Gene expression was assessed using PowerUp SYBR Green Master Mix (Thermo Fisher Scientific) and primers are listed in Supplemental Table 4. RNA levels were normalized to *Hprt* RNA levels using the 2^–ΔCt^ method.

### Statistics.

GraphPad Prism software was used for graphical presentation of quantitative data and statistical analyses. We ensured reproducibility by performing in vitro experiments at least 3 times, each with technical triplicates. For in vivo assays, we determined experimental group size (*n* ≥ 3) based on results from preliminary and published studies in the same field. Individual data points are shown, or *n* is indicated. Results are depicted as mean ± SD if not stated otherwise. Student’s 2-tailed *t* tests were used to compare data from 2 groups, and 1-way ANOVA followed by Tukey’s multiple-comparison tests from more than 2 groups. *P* values ≤ 0.05 were considered significant. Schematics were created using BioRender and Adobe Photoshop software.

### Study approval.

Mice were used as approved by the Children’s Hospital of Philadelphia Institutional Animal Care and Use Committee.

### Data availability.

Values for all data points in the graphs are reported in the Supporting Data Values file. All plasmids and mouse lines generated for this project or additional information required to analyze the reported data are available upon request.

## Author contributions

VL conceived the project. VL and MA supervised the project and wrote the manuscript. MA, ANM, AK, AFI, SW, and AB performed the experiments. All authors analyzed data and approved the manuscript.

## Supplementary Material

Supplemental data

Unedited blot and gel images

Supplemental table 3

Supplemental video 1

Supplemental video 2

Supplemental video 3

Supporting data values

## Version 1 01/16/2025

Electronic publication
