# Supplementary material for: Fgfr3 enhancer deletion markedly improves all skeletal features in a mouse model of achondroplasia
Source: J Clin Invest. 2025 Jan 16;135(2):e184929. doi: 10.1172/JCI184929 (PMC11735107; doi:10.1172/JCI184929)
Supplement: Supplemental data [file jci-135-184929-s110.pdf]

SUPPLEMENTAL MATERIALS

Supplemental figures

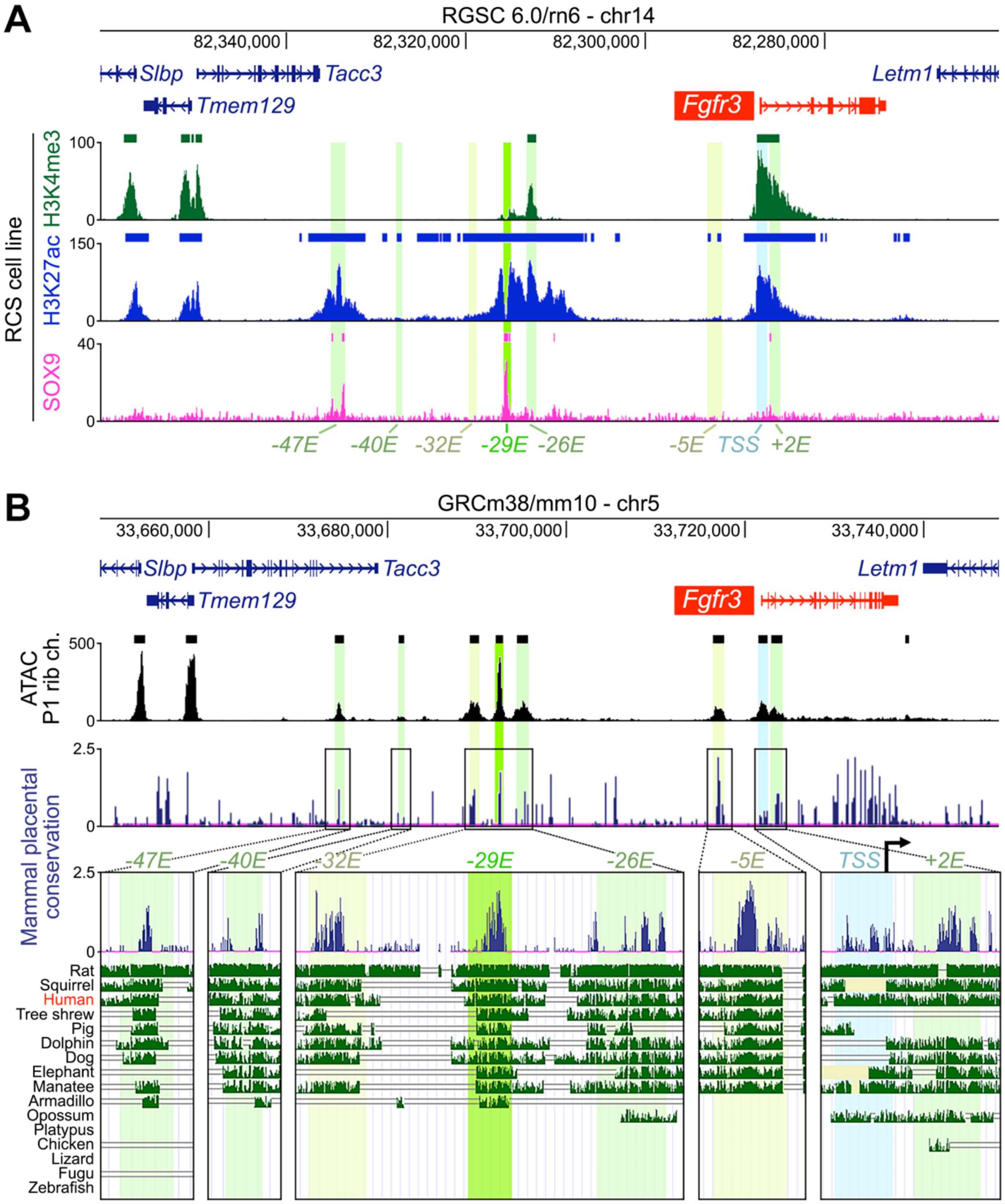

Supplemental Figure 1. Identification of putative enhancers of *Fgfr3* in chondrocytes. (**A**) Top, rat chromosome 14 segment containing *Fgfr3* and neighbor genes. Bottom, ChIP-seq data for H3K4me3, H3K27ac and SOX9 in RCS cells (*28*), with horizontal lines/blocks indicating peaks. Putative enhancers are highlighted in green shades and the *Fgfr3* transcription start site (TSS) in light blue. **(B)** Top, mouse chromosome 5 segment encompassing *Fgfr3* and neighbor genes; middle, ATAC-seq data from P1 mouse rib primary chondrocytes (*26*). Bottom, placental mammal conservation scores (UCSC Genome Browser). Boxed areas show enlarged histograms of conservation scores for putative enhancers and the *Fgfr3* promoter, with alignment tracks for representative species of mammals and other vertebrates.

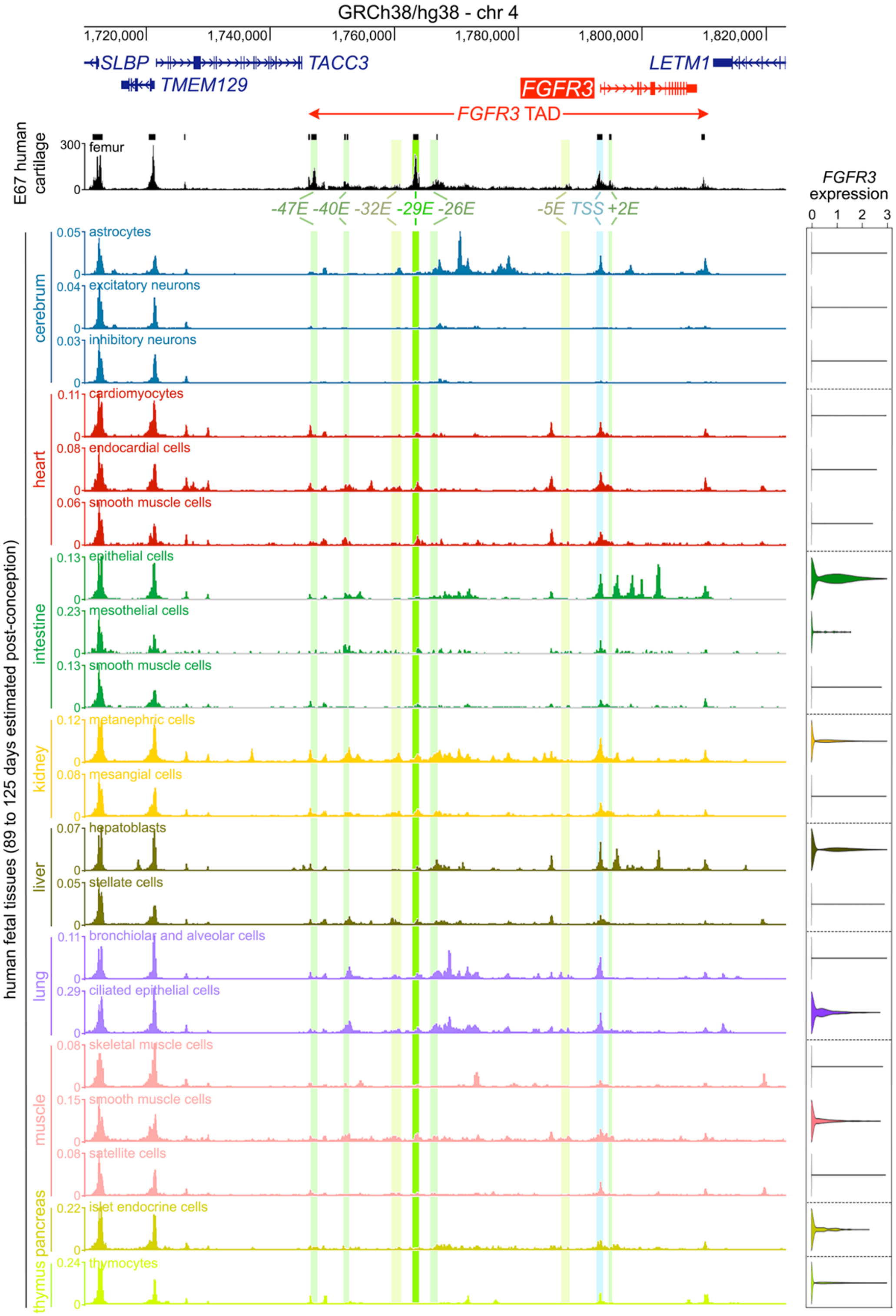

Supplemental Figure 2. Identification of putative enhancers of *Fgfr3* in non-skeletal tissues. Top, graphical representation of the human chromosome 4 segment containing *FGFR3*. Bottom, single-cell ATAC-seq data from various non-skeletal fetal cells (30). *FGFR3* RNA levels obtained through single-cell RNA-seq assays in the same samples are shown in violin plots on the right. Putative cartilage enhancers of *FGFR3* are highlighted in green shades and the *FGFR3* transcription start site (TSS) in light blue.

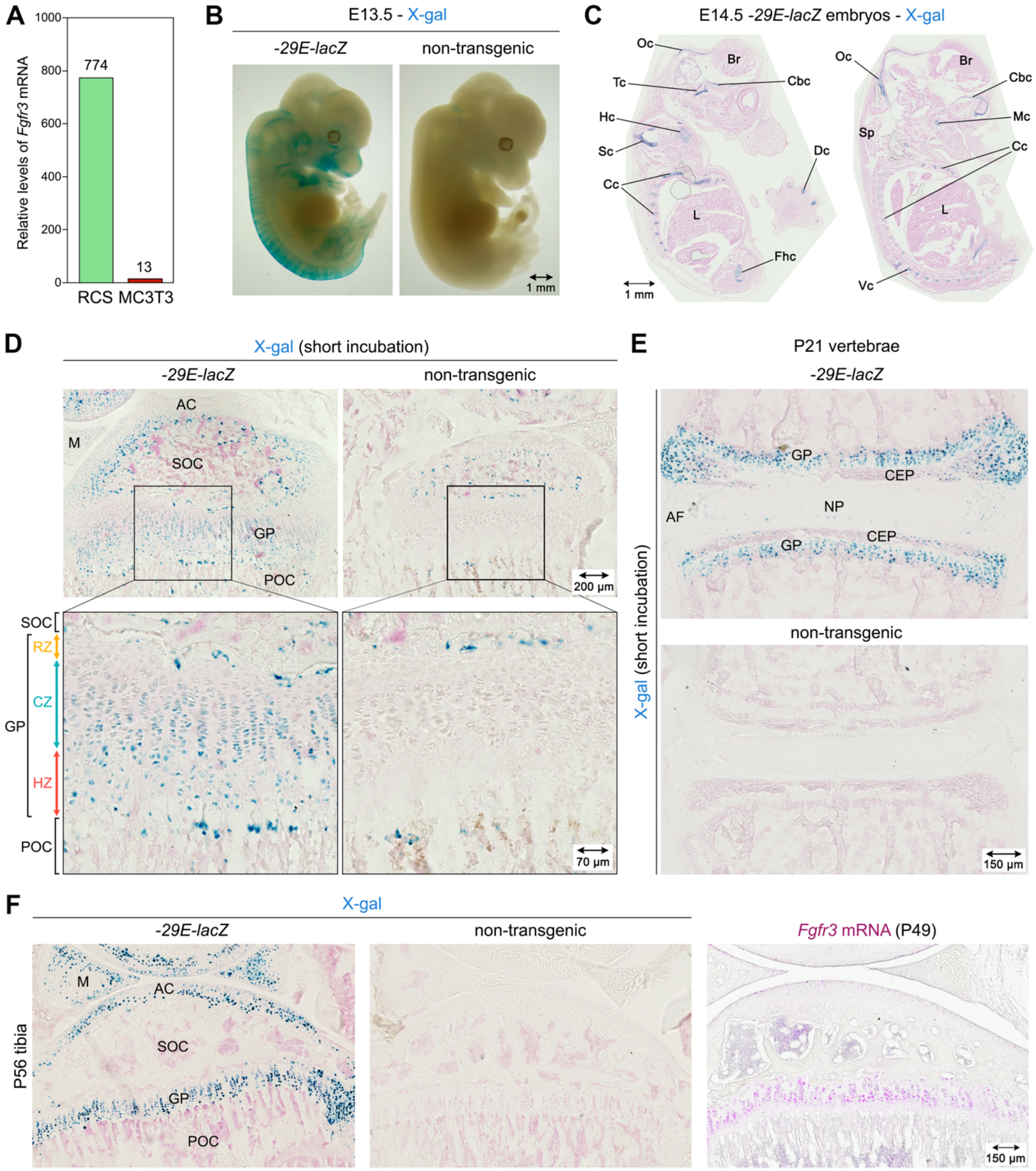

Supplemental Figure 3. *-29E* drives expression of a reporter transgene in cartilage. (**A**) RT-qPCR quantification of *Fgfr3* relative to *Hprt* RNA amounts in RCS and MC3T3-E1 cells. (**B**) Representative E13.5 *-29E-lacZ* and non-transgenic littermates stained with X-gal (blue). (**C**) Representative non-adjacent sagittal sections through an E14.5 *-29E-lacZ* embryo stained with X-gal and counterstained with Nuclear Fast Red (pink). Br, brain; Cbc, cranial base cartilage; Cc, costal cartilage; Dc, digital cartilage; Fhc, femoral head cartilage; Hc, humerus cartilage; L, liver; Mc, Meckel's cartilage; Oc, occipital cartilage; Sc, scapula cartilage; Sp, spinal cord; Tc, temporal cartilage; Vc, vertebral cartilage. (**D**) X-gal staining of representative sections through the tibia proximal GPs of P21 *-29E-lacZ* and non-transgenic mice. Sections

are adjacent to those shown in Fig. 2H but were stained for 5 h instead of overnight. Boxed areas are shown at high magnification below. AC, articular cartilage; M, meniscus; POC, primary ossification center; SOC, secondary ossification center. X-gal staining in ossification centers of transgenic and non-transgenic mice reflects endogenous β-galactosidase expressed in osteoclasts. (**E**) X-gal staining of representative coronal sections through the vertebral column of the same mice as in (D). These sections are adjacent to those shown in Fig. 2I but were stained for 5 h instead of overnight. AF, annulus fibrosus; CEP, cartilaginous end plate; NP, nucleus pulposus. (**F**) Left and middle, X-gal staining of representative sections through the tibia proximal GPs of P56 *-29E-lacZ* and non-transgenic littermates. Right, *Fgfr3* RNA in situ hybridization (magenta) of a section from a P49 wild-type mouse at a level equivalent to those used for X-gal staining. The magenta color (RNA signal) was saturated, and the blue color (hematoxylin) was desaturated using Adobe Photoshop.

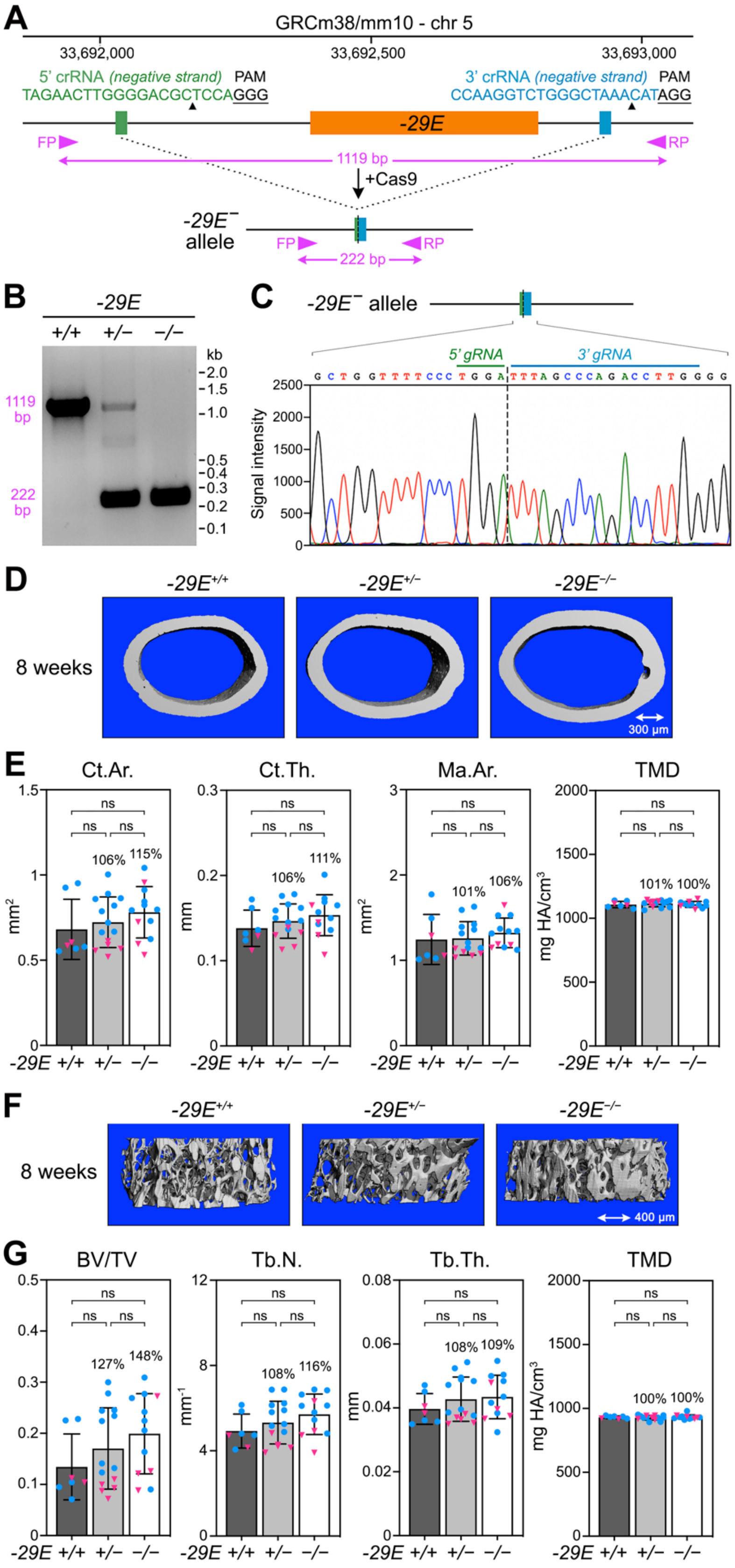

A
GRCm38/mm10 - chr 5
33,692,000
33,692,500
33,693,000
5' crRNA (negative strand) PAM
TAGAACTTGGGGACGCTCCAGGG
3' crRNA (negative strand) PAM
CCAAGGTCTGGGCTAAACATAGG
-29E
FP
RP
1119 bp
+Cas9
-29E⁻ allele
222 bp
B
-29E
+/+ +/− −/−
kb
1119 bp
222 bp
C
-29E⁻ allele
5' gRNA
3' gRNA
Signal intensity
D
-29E+/+
-29E+/−
-29E−/−
8 weeks
300 µm
E
Ct.Ar.
Ct.Th.
Ma.Ar.
TMD
ns
106%
115%
111%
101%
100%
mm²
mm
mg HA/cm³
-29E +/+ +/− −/−
F
8 weeks
400 µm
G
BV/TV
Tb.N.
Tb.Th.
TMD
127%
148%
108%
116%
109%
mm⁻¹

Supplemental Figure 4. Generation and genotyping of mice lacking *-29E*. (**A**) Graphical rendering of the *Fgfr3* allele containing *-29E* before (*-29E*$^{+}$) and after (*-29E*$^{-}$) its deletion by CRISPR/Cas9 technology. *-29E* is shown as an orange box. The crRNAs are shown with green and blue boxes. Their sequence, the adjacent PAM sequence, and the actual breaking point (arrowhead) are indicated. Pink arrowheads depict genotyping primers, and double arrows show amplicons and their size. A vertical dotted line in the *-29E*$^{-}$ allele schematic indicates the site of non-homologous end joining. (**B**) Image of electrophoresed PCR products revealing wild-type (*-29E*$^{+/+}$), heterozygous mutant (*-29E*$^{+/-}$), and homozygous mutant (*-29E*$^{-/-}$) mouse genotypes. (**C**) Representative chromatogram of Sanger sequencing of the PCR product obtained for the mutant allele of a *-29E*$^{+/-}$ mouse founder. The dotted line marks the site of non-homologous end joining. (**D**) µCT reconstruction images of femoral cortical bones from representative 8-week-old *-29E*$^{+/+}$, *-29E*$^{+/-}$ and *-29E*$^{-/-}$ mice. (**E**) Quantification of cortical bone area (Ct.Ar.), cortical thickness (Ct.Th.), marrow area (Ma.Ar.), and cortical bone mineral density (TMD) of femurs of mice with the same genotypes as in (D). Bars and brackets represent means and standard deviations, respectively. Each symbol represents a distinct mouse. Blue dots, males; pink triangles, females. The percentages of average values for each group relative to *-29E*$^{+/+}$ mice are indicated. The statistical significance of differences among genotypes was assessed using one-way ANOVA followed by Tukey's multiple comparison tests. No significant differences were detected among genotypes (ns). (**F**) µCT reconstruction images of trabecular bone in the femoral secondary spongiosa of representative mice from the same groups as in (D). (**G**) Quantification of bone volume/tissue volume (BV/TV), trabecular number (Tb.N.), thickness (Tb.Th.), and mineral density (TMD) of femurs of mice from the same groups as in (F).

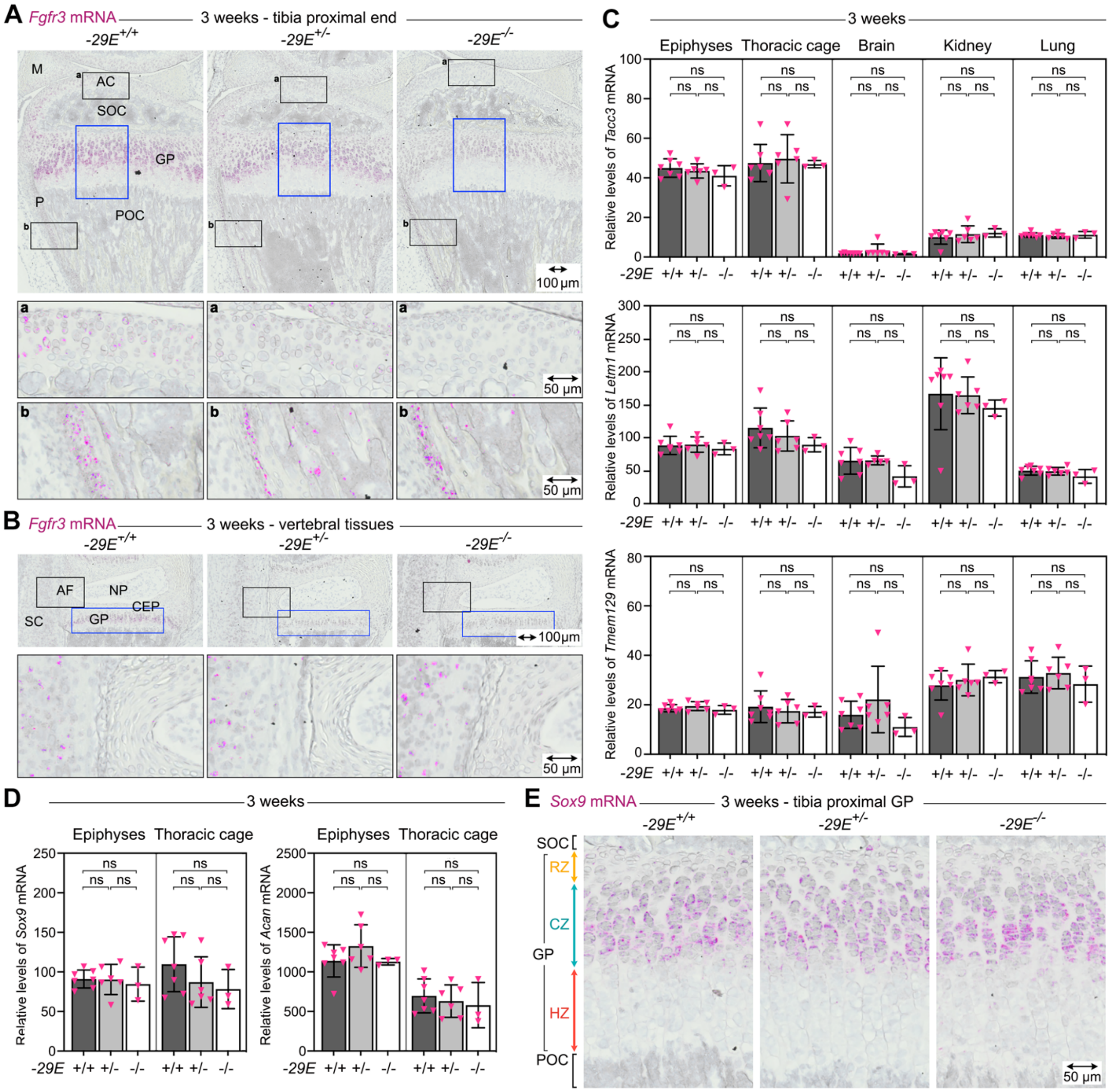

Supplemental Figure 5. *-29E* deletion lowers *Fgfr3* expression in cartilage tissues. (**A**) *Fgfr3* RNA in situ hybridization of sections through tibia proximal GPs of 3-week-old *-29E*$^{+/+}$, *-29E*$^{+/-}$ and *-29E*$^{-/-}$ mice. Data are representative of assays performed for three mice per genotype. Blue-boxed areas are shown at higher magnification in Figure 3I. Black-boxed areas containing articular cartilage (a) or periosteum (b) are shown at higher magnification below. Adobe Photoshop was used to saturate the magenta color (RNA signal) and desaturate the blue color (hematoxylin). AC, articular cartilage; M, meniscus; P, periosteum; POC, primary ossification center; SOC, secondary ossification center. (**B**) *Fgfr3* RNA in situ hybridizations of sections through the vertebral column of the same mice as in (A). Blue-boxed areas are shown at higher magnification in Figure 3J. Black-boxed areas are shown at higher magnification below. Images were processed as in (A). AF, annulus fibrosus; CEP, cartilaginous end plate; NP, nucleus pulposus; SC, spinal cord. (**C**) RT-qPCR assays of the RNA amounts of *Fgfr3* neighbors (*Tacc3*, *Letm1* and *Tmem129*) in skeletal and non-skeletal tissues from 3-week-old *-29E*$^{+/+}$, *-29E*$^{+/-}$ and *-29E*$^{-/-}$ female mice. Bars and

brackets represent means and standard deviations, respectively. Each symbol corresponds to a distinct mouse. The statistical significance of differences among genotypes was assessed using one-way ANOVA followed by Tukey's multiple comparison tests. No significant differences were detected among genotypes (ns). (**D**) RT-qPCR assays of the *Sox9* and *Acan* RNA levels in skeletal tissues from the same mice as in (C). (**E**) *Sox9* RNA in situ hybridization of tibia proximal GP sections adjacent to those shown in (A). Images were processed similarly.

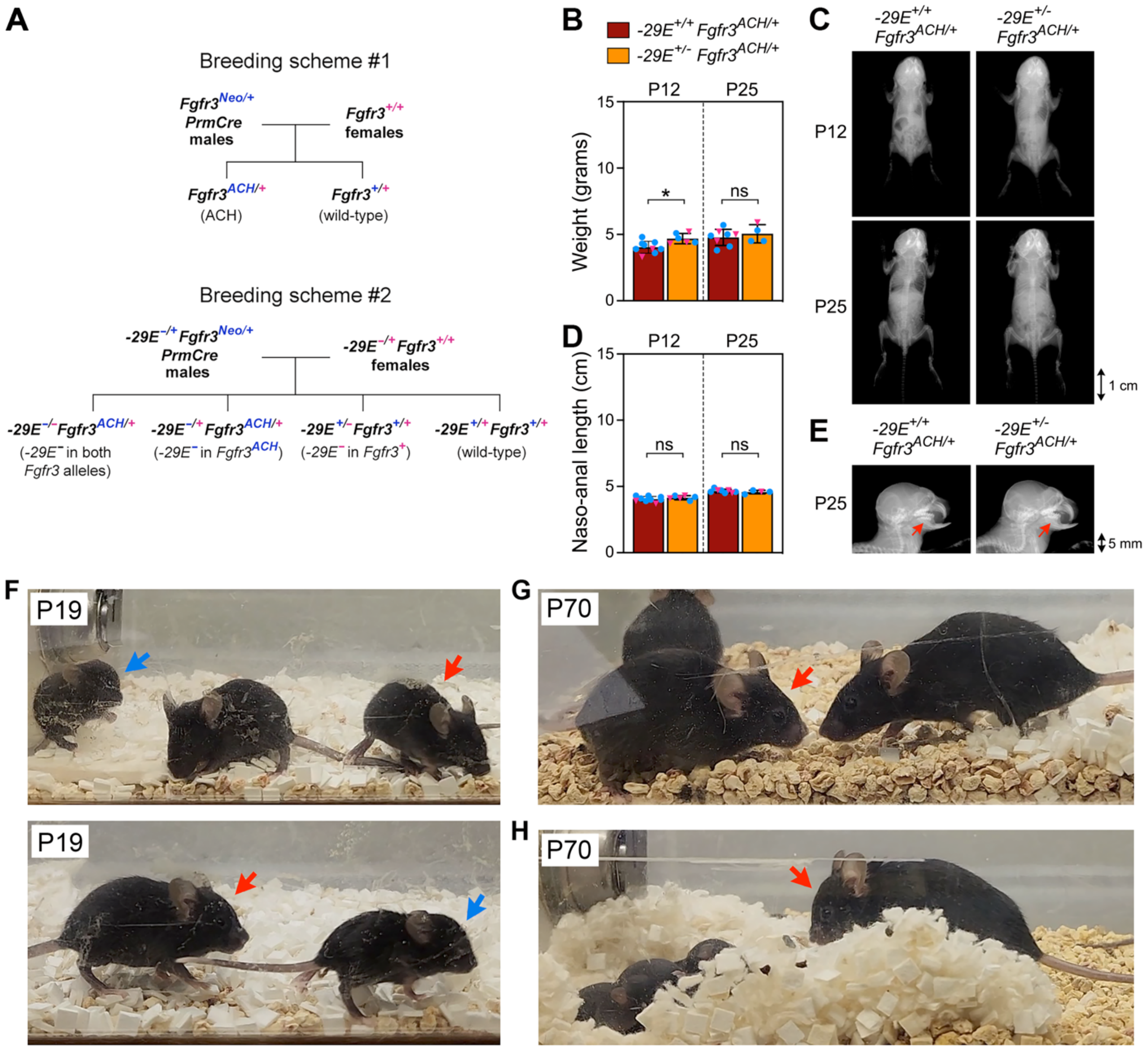

Supplemental Figure 6. Breeding strategies and absence of effect of *-29E* deletion in the wild-type *Fgfr3* allele of achondroplasia mice. (**A**) Breeding strategies to obtain experimental mice with or without *-29E* deletion in the *Fgfr3* wild-type and *ACH* alleles (relevant to Figures 4-7 and S7-S9). The first strategy allowed to generate ACH and wild-type littermates with intact -29E in both *Fgfr3* alleles. The second strategy allowed to generate ACH mice with *-29E* deletion in the ACH allele only or in both *Fgfr3* alleles, and control mice wildtype or heterozygous for -29E deletion. Of note, the *PrmCre* transgene used to recombine $Fgfr3^{Neo}$ into $Fgfr3^{ACH}$ in the germline of male breeders is not indicated for progeny because it

does not influence phenotypes. (**B**) Weights of P12 and P25 achondroplasia mice without or with *-29E* deletion in the *Fgfr3* wild-type allele. Bars and brackets represent means and standard deviations, respectively. Each symbol represents a distinct mouse. Blue dots, males; pink triangles, females. The statistical significance of differences was assessed by two-tailed unpaired Student's t tests (ns, non-significant; *, p ≤0.05). (**C**) X-ray images of representative mice from the same groups as in (B). (**D**) Naso-anal lengths of the same mice as in (B). (**E**) X-ray images of the skulls of representative P25 mice from the same groups as in (B). Red arrows indicate jaw misalignment. (**F**) Pictures of representative P19 wildtype mice ($-29E^{+/+}Fgfr3^{+/+}$), achondroplasia mice ($-29E^{+/+}Fgfr3^{ACH/+}$) and achondroplasia mice with *-29E* deletion in both *Fgfr3* alleles ($-29E^{-/-}Fgfr3^{ACH/+}$) shown in movie S1. Note that the $-29E^{-/-}Fgfr3^{ACH/+}$ mouse (red arrow) has a size intermediate between those of the ACH mouse (blue arrow) and control mouse. (**G**) Picture of a representative $-29E^{-/+}Fgfr3^{ACH/+}$ male (red arrow) with a wildtype female, both at P70, shown in movie S2. Note that the male has a flatter head than the female, but looks healthy. (**H**) Picture of a representative P70 $-29E^{-/+}Fgfr3^{ACH/+}$ female (red arrow) with her litter (P7) shown in movie S3.

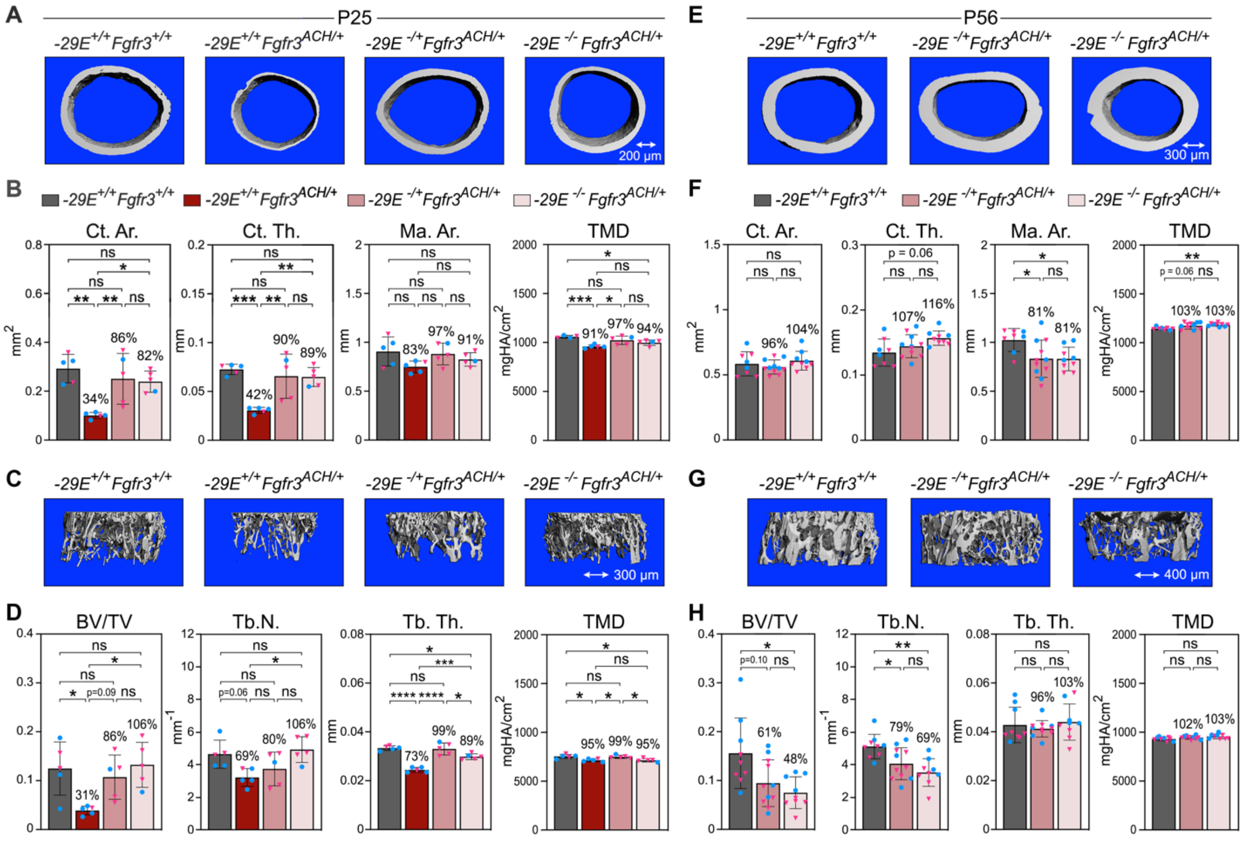

Supplemental Figure 7. *-29E* deletion improves bone parameters in achondroplasia mice. (**A**) µCT reconstruction images of femoral cortical bones from representative P25 wild-type mice ($-29E^{+/+}Fgfr3^{+/+}$), achondroplasia mice ($-29E^{+/+}Fgfr3^{ACH/+}$) and achondroplasia mice with *-29E* deletion in the ACH allele ($-29E^{-/+}Fgfr3^{ACH/+}$) or in both alleles ($-29E^{-/-}Fgfr3^{ACH/+}$). (**B**) Quantification of cortical bone area (Ct.Ar.), cortical thickness (Ct.Th.), marrow area (Ma.Ar.), and cortical bone mineral density (TMD) of femurs of mice with the same genotypes and age as in (A). Bars and brackets represent means and standard deviations, respectively. Each symbol represents a distinct mouse. Blue dots, males; pink triangles, females. The

percentages of average values for each group relative to *-29E*$^{+/+}$*Fgfr3*$^{+/+}$ mice are indicated. The statistical significance of differences among genotypes was assessed using one-way ANOVA followed by Tukey's multiple comparison tests (ns, non-significant; *, p ≤0.05; **, p ≤0.01; ***, p ≤0.001; ****, p ≤0.0001). (**C**) µCT reconstruction images of trabecular bones in the femoral primary spongiosa of representative mice from the same groups as in (A). (**D**) Quantification of bone volume/tissue volume (BV/TV), trabecular number (Tb.N.), thickness (Tb.Th.), and mineral density (TMD) of femurs of mice from the same groups as in (B). (**E**) µCT reconstruction images of femoral cortical bones from representative 8-week-old *-29E*$^{+/+}$*Fgfr3*$^{+/+}$, *-29E*$^{-/+}$*Fgfr3*$^{ACH/+}$ and *-29E*$^{-/-}$*Fgfr3*$^{ACH/+}$ mice. (**F**) Quantification of the cortical bone parameters of femurs of mice with the same genotypes and age as in (E). (**G**) µCT reconstruction images of trabecular bones in the femoral secondary spongiosa of representative mice from the same groups as in (E). (**H**) Quantification of the trabecular bone parameters of femurs of mice from the same groups as in (E).

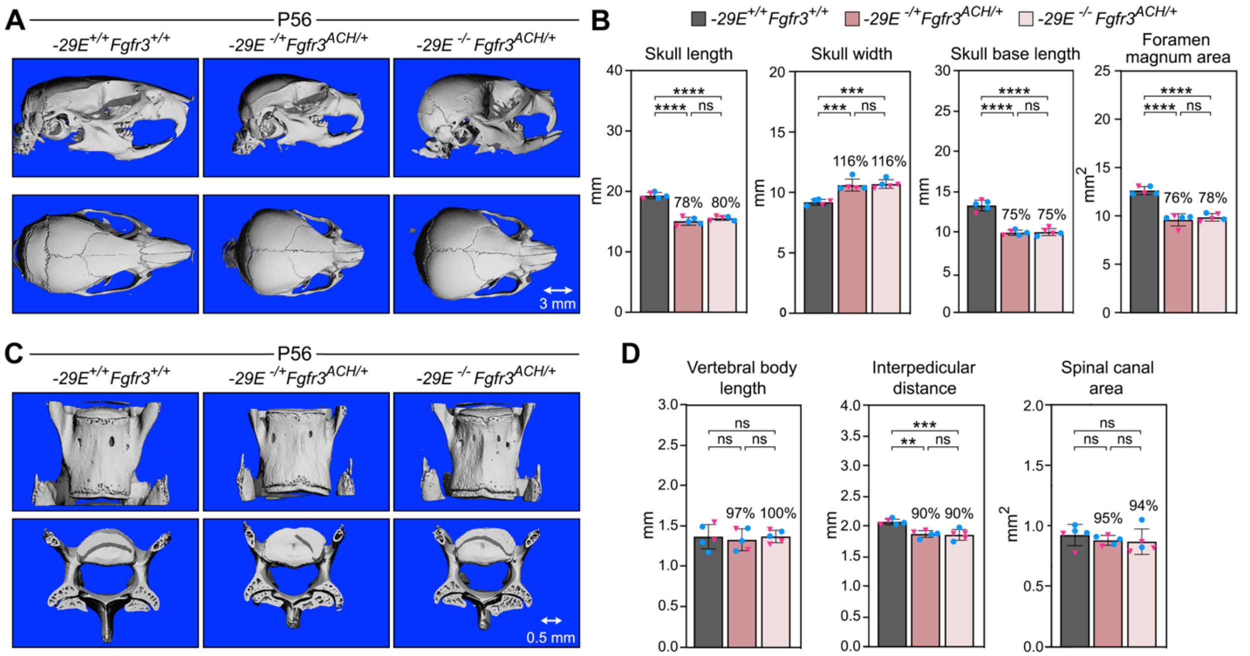

Supplemental Figure 8. *-29E* deletion lessens skull and vertebral defects of achondroplasia mice. (**A**) µCT reconstruction images of skulls from representative P56 mice from the same groups as in (B). Top row, lateral view; bottom row, apical view. (**B**) Skull length, width, base length, and foramen magnum area measurements of mice with the indicated genotypes. Bars and brackets represent means and standard deviations, respectively. Each symbol corresponds to a distinct mouse. Blue dots, males; pink triangles, females. The percentages of average values for each genotype group relative to *-29E*$^{+/+}$*Fgfr3*$^{+/+}$ mice are indicated. The statistical significance of differences among genotypes was assessed by one-way ANOVA followed by Tukey's multiple comparison tests (ns, non-significant; *, p ≤0.05; **, p ≤0.01; ***, p ≤0.001; ****, p ≤0.0001). (**C**) µCT reconstruction images of the L4 vertebrae of the same mice as in (B). Top row, coronal view; bottom row, transverse view. (**D**) Vertebral body length, interpedicular distance and canal area measurements of L4 vertebrae from the same mice as in (B).

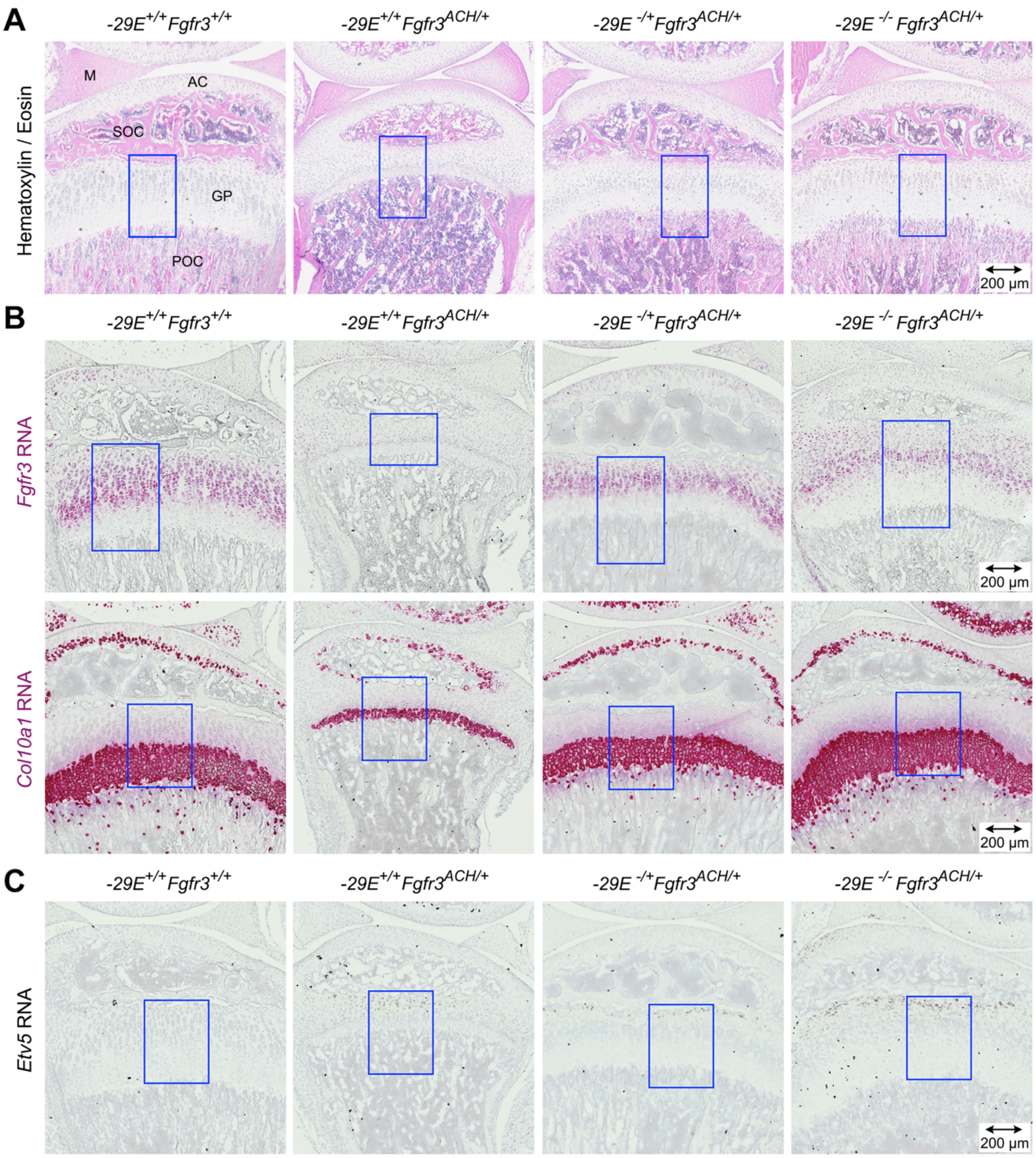

Supplemental Figure 9. *-29E* deletion lessens GP defects of achondroplasia mice. (**A**) Pictures of hematoxylin-stained sections through the tibia proximal GPs of representative P25 mice with the indicated genotypes. Blue-boxed areas are shown at higher magnification in Figure 6A. AC, articular cartilage; M, meniscus; POC, primary ossification center; SOC, secondary ossification center. (**B**) *Fgfr3* and *Col10a1* RNA in situ hybridization in sections through the tibia proximal GPs of the same mice as in (A). RNA signals are shown in magenta. The blue color resulting from hematoxylin counterstaining was desaturated using Adobe Photoshop. Blue-boxed areas are shown at higher magnification in Figure 6E. (**C**) *Etv5* RNA in situ hybridization in sections through the proximal tibial GPs of the same mice as in (A). Blue-boxed areas are shown at higher magnification in Figure 7C. RNA signals (dark brown) were saturated, and the blue color (hematoxylin) was desaturated using Adobe Photoshop.

Supplemental tables

Supplemental Table 1. List of primers used to generate reporter plasmids containing putative *Fgfr3* enhancers.

Target	Oligonucleotide sequence	Amplicon size
Fgfr3 (-345/+591)	FP with PstI restriction site: CTG CAG CAT TCA GCT CAG GGG GCC AGT TCA CTG<br>RP with HindIII restriction site: AAG CTT CAA GCA GCG CGC GCG GGC ATG CTG CAA	956 bp
-47E	FP with BamHI restriction site: GAT TGG ATC CTA GAG CTG CCC TAT CTA TCC CTT AC<br>RP with BglII restriction site: CTG GAG ATC TTG CCT CAG GGC ATT CCC TAC TTT G	388 bp
-29E	FP with XhoI and SpeI restriction sites: GAT TCT CGA GAC TAG TAG GTT CAG GGG TGT GGC TGA CTG CT<br>RP with PstI and NheI restriction sites: CAT GCT GCA GGC TAG CGT TGG GGC AAG GGT GAG GCC ATG	593 bp
-26E	FP with BamHI restriction site: GAT TGG ATC CTG TTA GTC GCC CCC TCC CGG GGC TT<br>RP with BglII restriction site: GAC CAG ATC TAG GGG TTG GCC GCG TGG GGC GT	496 bp
+2E	FP with BamHI restriction site: GAT TGG ATC CGC AGC GGT CTG GCG GGA GGC CGC GCG A<br>RP with BglII restriction site: GAC CAG ATC TCG ACG CAA GAA GTA ATA TCT TCA TCT GAA TAC AC	486 bp

Supplemental Table 2. List of primers used to genotype *-29E-lacZg*, *-29E*$^{+/-}$, *Fgfr3*$^{Neo/+}$ and *Fgfr3*$^{Ach/+}$ mice.

Target	Oligonucleotide sequence	Amplicon size
lacZ	FP: AGT TGA GGC TGA CAC TGT TGT G<br>RP: GTC TCT GAG TTC TCC ACA CAT CTG	522 bp
-29E^{+} and *-29E*$^{-}$	FP: ATT CAG CCA TGC CTT TTG TGT A<br>RP: CTA GAC CCA CCA GGC TGA AC	*-29E*$^{+}$: 1119 bp<br>*-29E*$^{-}$: 222 bp
Fgfr3^{+}, *Fgfr3*^{Neo} and *Fgfr3*^{Ach}	FP: GTA GAG TGT AGT GAG GTC ACT GCA<br>RP: AGC TGT AAC CAG TGC CTT CC	*Fgfr3*$^{+}$: 440 bp<br>*Fgfr3*^{Neo}: 1500 bp<br>*Fgfr3*^{Ach}: 224 bp

Cre	FP: TGA GGT TCG CAA GAA CCT GAT GGA<br>RP: GCC GCA TAA CCA GTG AAA CAG CAT	293 bp

Supplemental Table 3. Off-target analysis for crRNAs used to delete *-29E* in *Fgfr3*$^{+/+}$ and *Fgfr3*$^{Neo/+}$ mice. See Excel sheet file.

Supplemental Table 4. List of primers used for qRT-PCR assays. FP, forward primer; RP, reverse primer.

**Gene**	**Oligonucleotides**	**Assay ID**
Fgfr3 (mouse)	FP: CTG AAG CAC GTG GAA GTG AA<br>RP: GAC AGA ACC TCT AGC TCC TTG	Mm.PT.58.12297329 (Integrated DNA Technologies)
Tacc3 (mouse)	FP: GTG GTT CAA ATG GCA GCT G<br>RP: AGA CTT GTG GAA GCT GAA AGG	-
Letm1 (mouse)	FP: CAC CCC TTC CTC AGA AAT CAG<br>RP: AGC TTC AGT CCA TTG GCA C	-
Tmem129 (mouse)	FP: TGG CTG CAC TTC GGA TTG<br>RP: CTC GCC AGA CTC AAA CCT AC	-
Sox9 (mouse)	FP: TCC ACG AAG GGT CTC TTC TC<br>RP: AGG AAG CTG GCA GAC CAG TA	-
Acan (mouse)	FP: CCT TGT CAC CAT AGC AAC CT<br>RP: CTA CAG AAC AGC GCC ATC A	Mm.PT.58.10174685 (Integrated DNA Technologies)
Hprt (mouse)	FP: CCT CAT GGA CTG ATT ATG GAC AG<br>RP: TCA GCA AAG AAC TTA TAG CCC C	-
Fgfr3 (rat)	FP: GTG GCA TCA TCC TTT CAG CAT<br>RP: CCC CAA GTG GGA GCT ATC C	-
Hprt (rat)	FP: GCT TTT CCA CTT TCG CTG ATG<br>RP: GGT GAA AAG GAC CTC TCG AAG	Rn.PT.39a.22214832 (Integrated DNA Technologies)

Supplemental movies

Supplemental Movie 1. Video of representative P19 wildtype mice (*-29E*$^{+/+}$*Fgfr3*$^{+/+}$), achondroplasia mice (*-29E*$^{+/+}$*Fgfr3*$^{ACH/+}$) and achondroplasia mice with *-29E* deletion in both *Fgfr3* alleles (*-29E*$^{-/-}$*Fgfr3*$^{ACH/+}$). Note that the *-29E*$^{-/-}$*Fgfr3*$^{ACH/+}$ mouse has a size intermediate between those of the ACH and control mice.

Supplemental Movie 2. Video of a representative P70 achondroplasia male mouse with *-29E* deletion in the ACH allele (*-29E*$^{-/+}$*Fgfr3*$^{ACH/+}$) with two wildtype (*-29E*$^{+/+}$*Fgfr3*$^{+/+}$) females of the same age. The male is in the center of the image at the start of the movie.

Supplemental Movie 3. Video of a representative P70 achondroplasia female with *-29E* deletion in the ACH allele (*-29E*$^{-/+}$*Fgfr3*$^{ACH/+}$) with a litter of P7 pups and a wildtype (*-29E*$^{+/+}$*Fgfr3*$^{+/+}$) male partner of the same age. The female is the mouse attending to the pups.
