# Supplementary figures and images for: Fgfr3 enhancer deletion markedly improves all skeletal features in a mouse model of achondroplasia
Source: J Clin Invest. 2025 Jan 16;135(2):e184929. doi: 10.1172/JCI184929 (PMC11735107; doi:10.1172/JCI184929)

# Full unedited gel for Figure S4

Box shows region selected for figure

-29E PCR

+/+    +/-    -/-    -/-    +/-    +/-

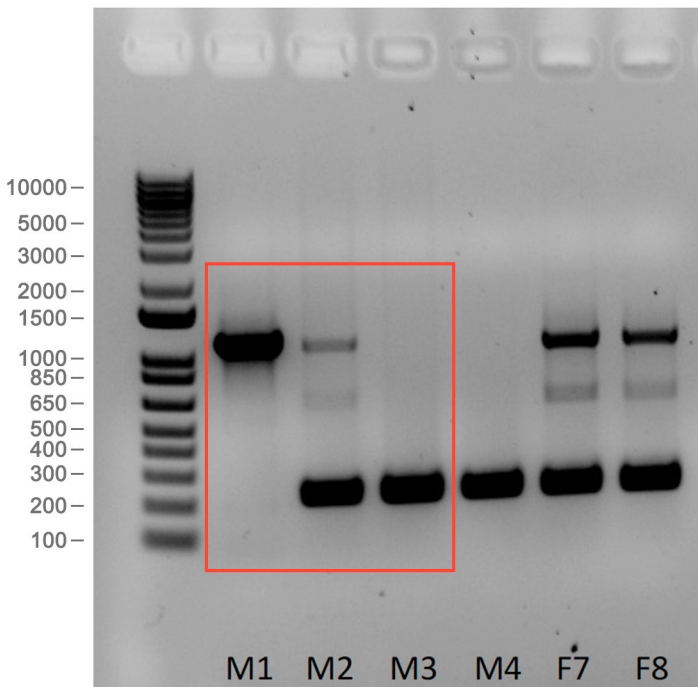

Supplement: Unedited blot and gel images [file jci-135-184929-s111.pdf]
